# Supplementary material for: Structural basis for inhibition of the lysosomal two-pore channel TPC2 by a small molecule antagonist
Source: Structure. 2024 Aug 8;32(8):1137–1149.e4. doi: 10.1016/j.str.2024.05.005 (PMC11511679; doi:10.1016/j.str.2024.05.005)
Supplement: Document S1. Figures S1–S6, Tables S1, and S2 [file mmc1.pdf]

Structure, Volume *32*

Supplemental Information

Structural basis for inhibition of the lysosomal two-pore channel TPC2 by a small molecule antagonist

Gamma Chi, Dawid Jaślan, Veronika Kudrina, Julia Böck, Huanyu Li, Ashley C.W. Pike, Susanne Rautenberg, Einar Krogsaeter, Tina Bohstedt, Dong Wang, Gavin McKinley, Alejandra Fernandez-Cid, Shubhashish M.M. Mukhopadhyay, Nicola A. Burgess-Brown, Marco Keller, Franz Bracher, Christian Grimm, and Katharina L. Dürr

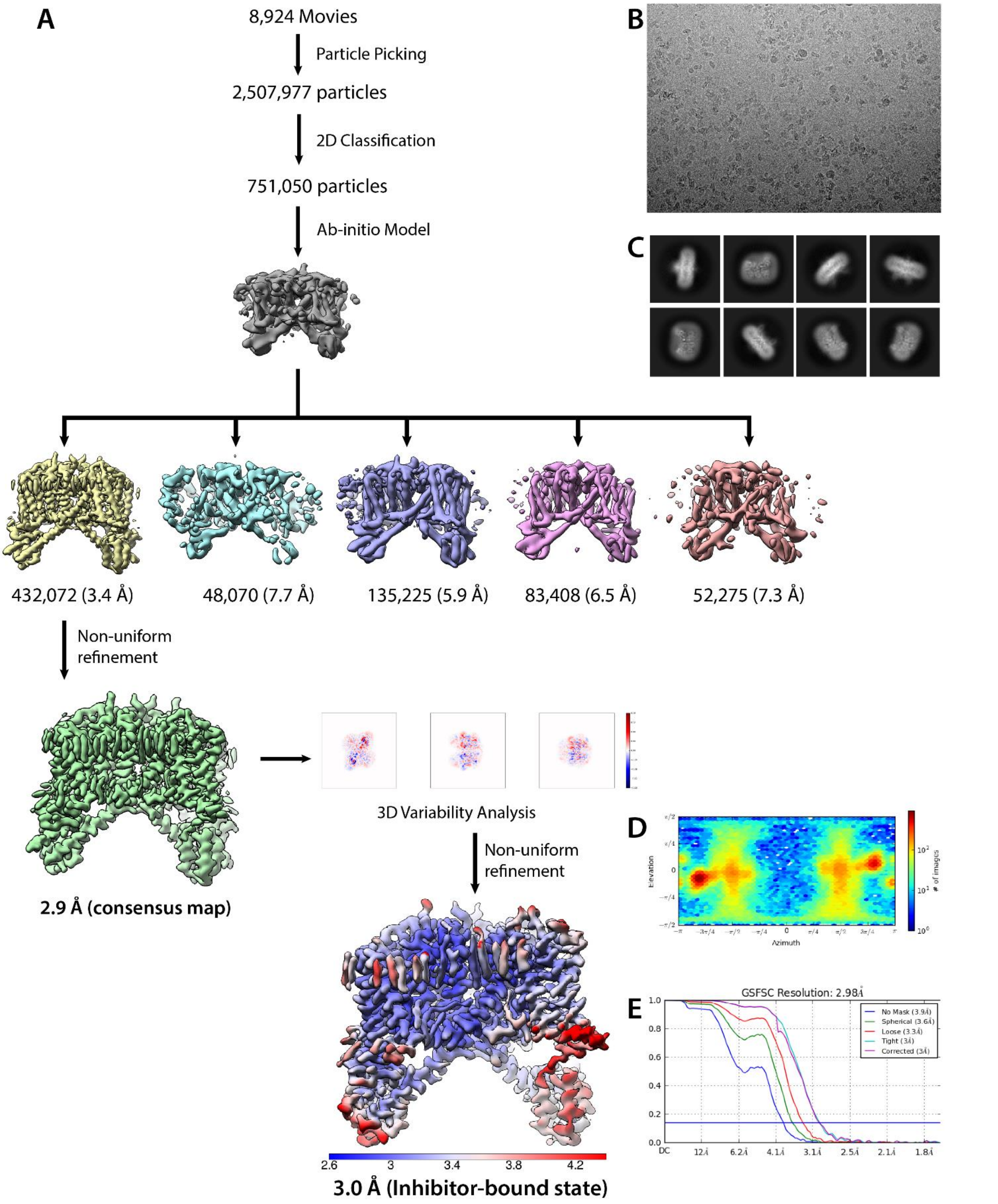

Supplementary Figure S1. Flowchart of structure determination with cryo-electron microscopy for *Hs*TPC2 in complex with small molecule antagonist *(S)*-SG-094, related to Figure 1 and STAR Methods (Cryo-EM Sample Preparation, Data Collection and Processing).

A) Data processing workflow. **B)** Sample raw micrograph of the collected dataset. **C)** Representative 2D classes of the collected dataset. **D)** Orientation distribution of the final reconstruction. **E)** Fourier Shell Correlation (FSC) curve of the final reconstruction

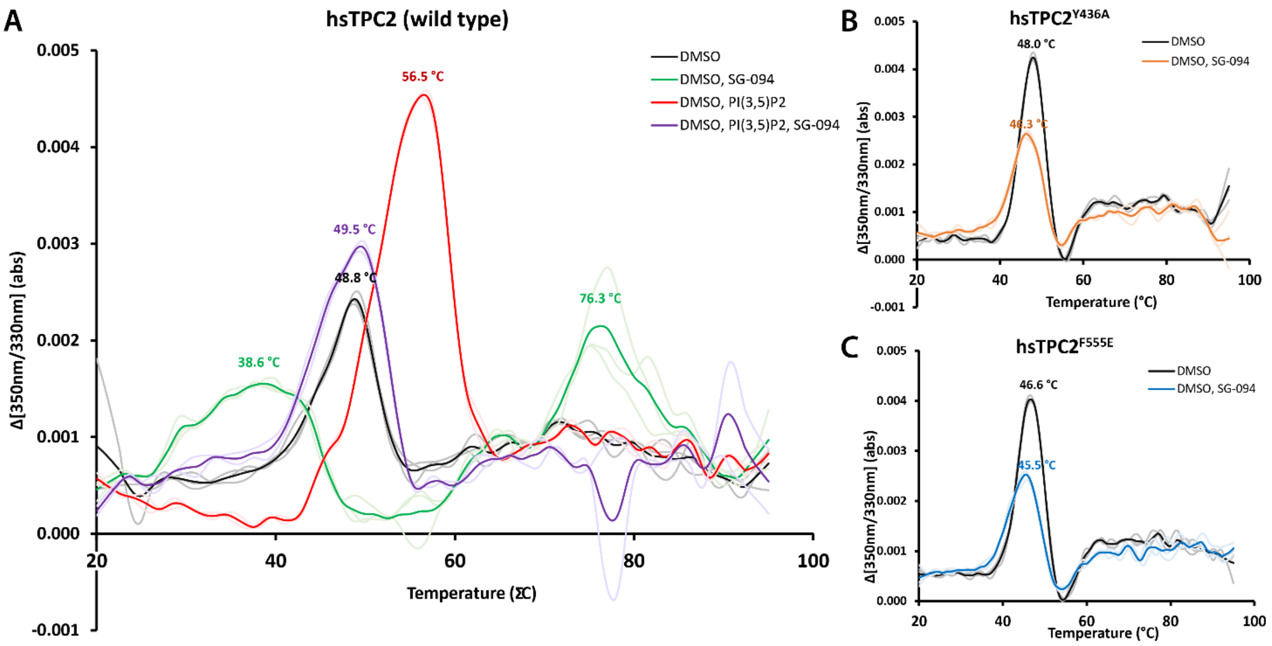

Supplementary Figure S2. Loss of *Hs*TPC2 stabilization by SG-094 for binding site mutants Y436A and F555E characterized in thermal shift experiments, related to Figures 4 and 5.

A– C) Thermal shift assay of *Hs*TPC2^{WT} (**A**) shows significant differences in melting profiles between apo (black trace) and SG-094-bound (green trace), suggesting that the compound binding alters the melting behavior of purified *Hs*TPC2. It also shows that the PI(3,5)P_2-mediated increase in thermostability (red trace) reversed to apo-like level (purple). In contrast, *Hs*TPC2^{Y436A} (**B**) and *Hs*TPC2^{F555E} (**C**) show little difference between apo state samples and SG-094-bound samples, indicating significantly reduced binding of the antagonist to *Hs*TPC2 with these mutations.

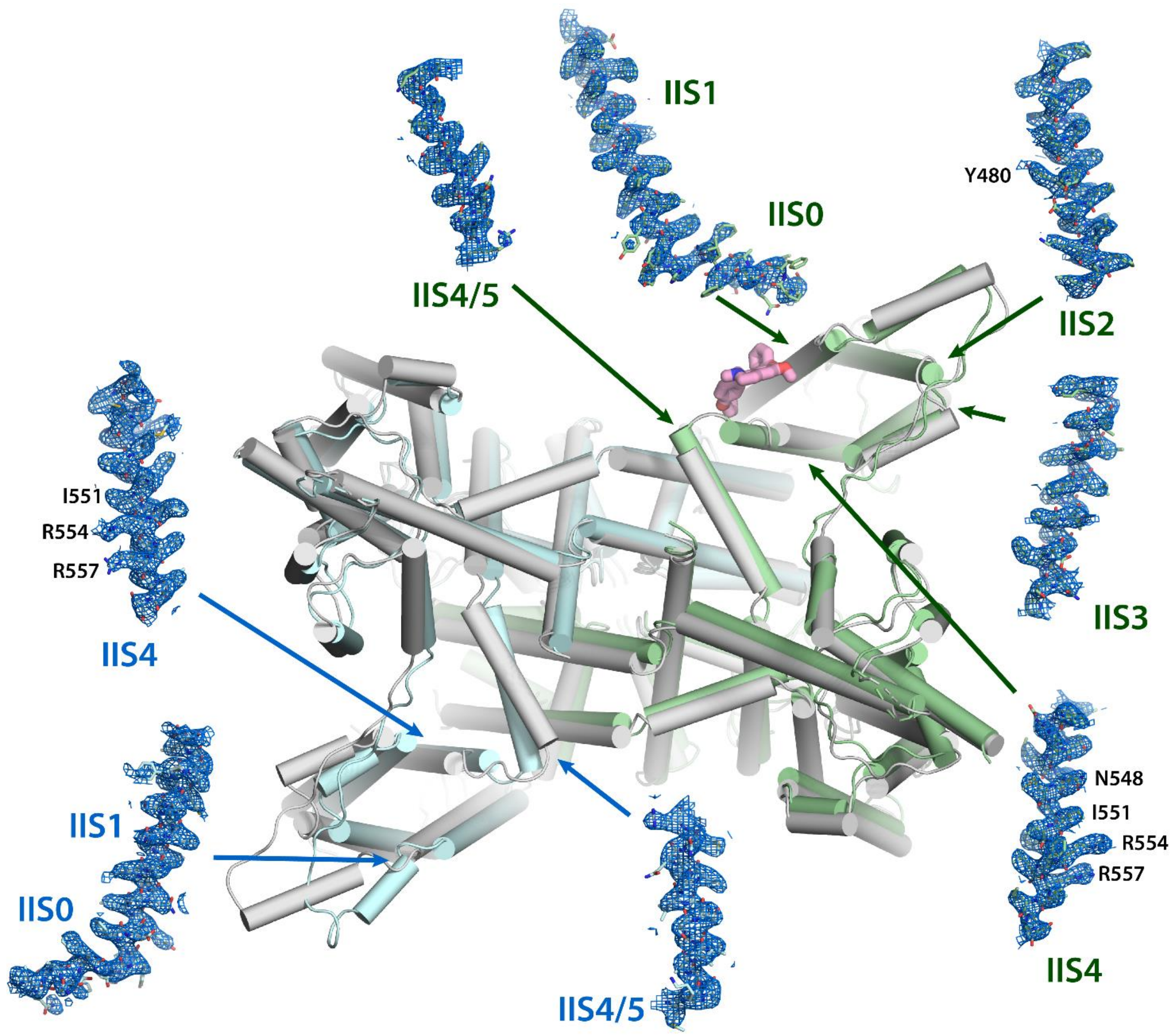

Supplementary Figure S3. Electron map features for key sites for the structure of HsTPC2 in complex with *(S)*-SG-094, related to Figures 1, 2, 6 and 7.

All electrostatic potential (ESP) map features have been contoured at 6.0 σ for consistency.

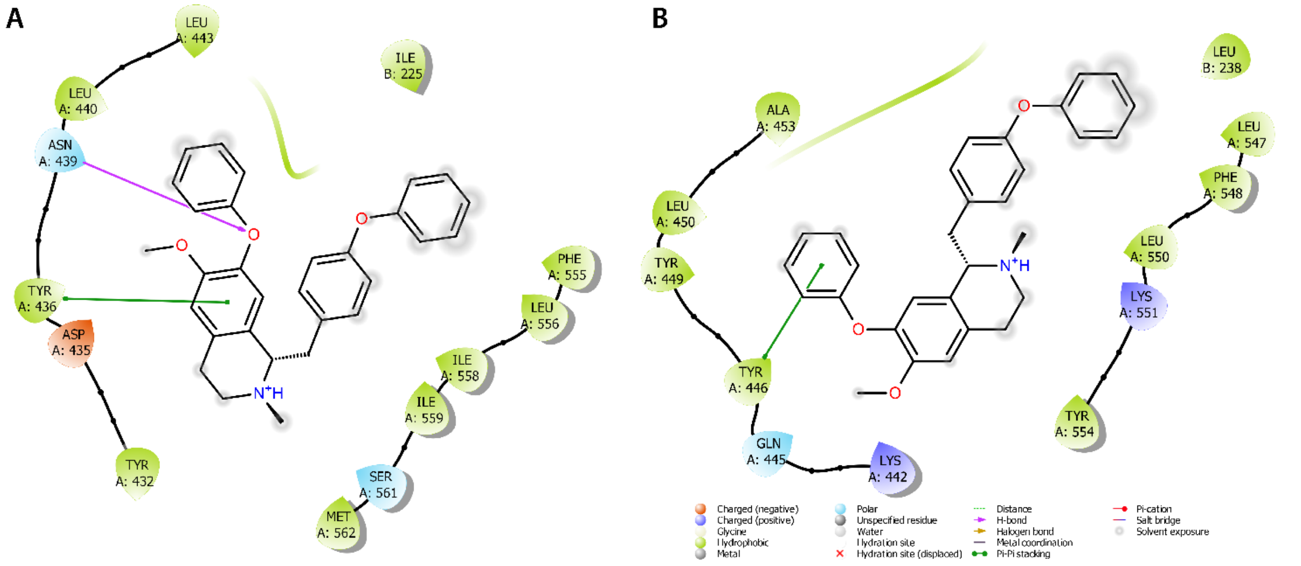

Supplementary Figure S4. Comparison of SG-094 interactions with *Hs*TPC2 to putative interactions of the antagonist bound to *Mm*TPC1, related to Figure 1.

A) Schematic of molecular interactions between SG-094 antagonist and residues in *Hs*TPC2 as inferred from the cryo-EM structure. **B)** Schematic of putative molecular interactions between SG-094 antagonist and the corresponding residues in *Hs*TPC1 as inferred from docking into the analogous binding site of *Mm*TPC1 (PDB ID: 6C96).

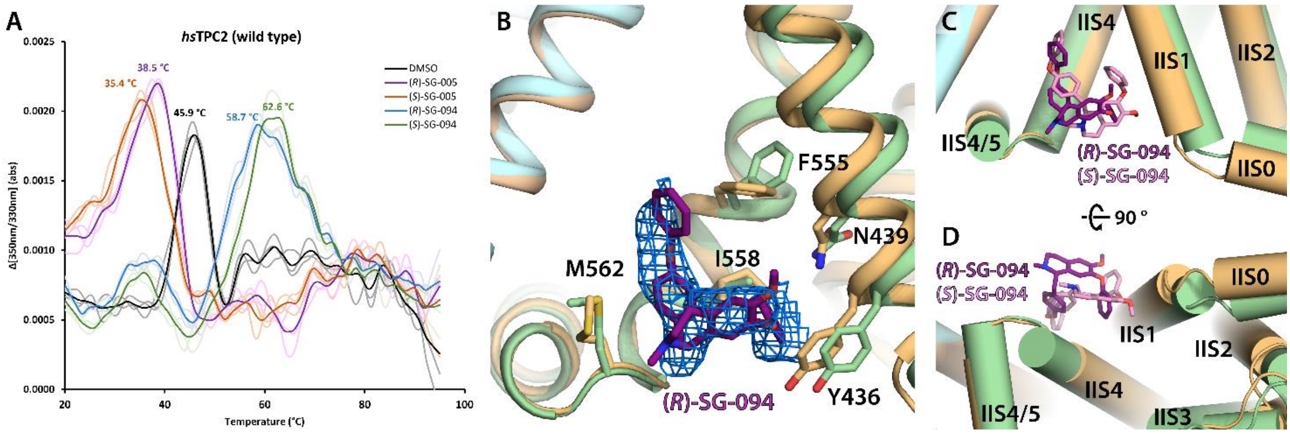

Supplementary Figure S5. Comparison of *(S)*-SG-094 and *(R)*-SG-094 interactions with *Hs*TPC2 showing similar biophysical properties and binding location, related to Figure 1.

A) Thermal shift experiments of SG-094 and SG-005 enantiomers show similar levels of stabilisation between the enantiomers of each compound. **B)** Cryo-EM electrostatic potential map of *Hs*TPC2 with *(R)*-SG-094 (σ=4.5) shows small compound feature at the same binding site as *(S)*-SG-094, however the level of fit is not good enough for modelling with confidence. Key residues are positioned similarly between *(R)*-SG-094 (light brown) and *(S)*-SG-094 (light green) bound *Hs*TPC2, suggesting similar effects of the compounds on the protein conformation. **C)** Comparison of *(S)*-SG-094 and *(R)*-SG-094 models shows near-identical binding site but with differences in binding mode.

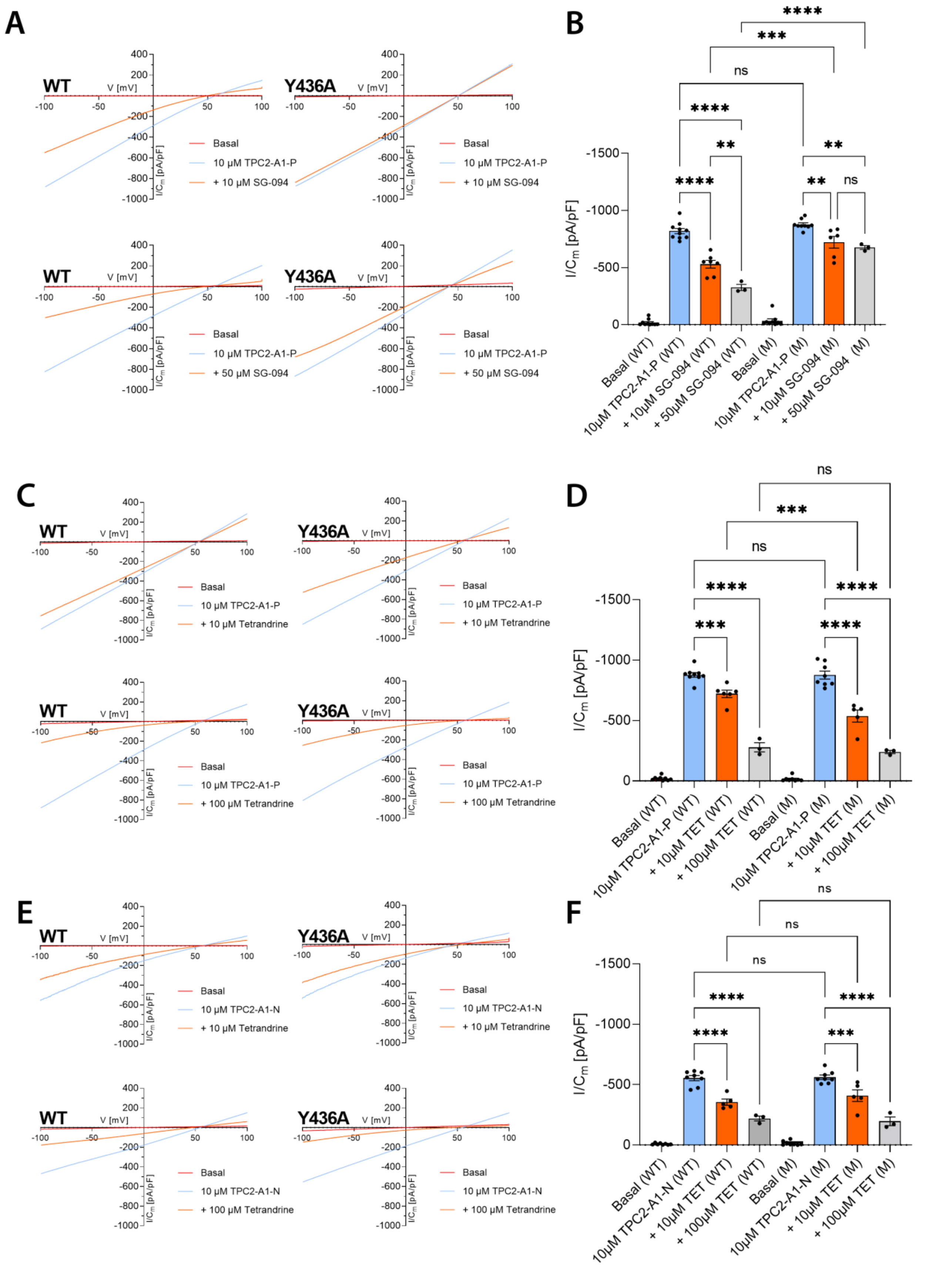

Supplementary Figure S6. TPC2-A1-P or TPC2-A1-N evoked hTPC2 current inhibited by SG-094 or tetrandrine for binding site mutant Y436A in whole cell patch-clamp recordings, related to Figures 4 and 5.

Representative current density-voltage (I/Cm-V) relation of transiently expressed, plasma membrane-targeted *Hs*TPC2$^{L11A/L12A}$-eYFP variants, WT (A, C and E – left) and Y436A mutant (A,

C and E – right). Channels were activated by application of TPC2-A1-P or TPC2-A1-N (10 µM, blue traces), followed by application of the TPC antagonists SG-094 (A; 10 µM and 50 µM, orange traces) tetrandrine (C, E; 10µM and 100 µM, orange traces). Basal current density was depicted with red color. Statistical analysis of experiments is shown in B, D, and F, with each dot representing mean of 5 – 10 technical replicate measurements (mean ± SEM; n = number of dots on the graph; one-way ANOVA, Tukey's post hoc test using GraphPad Prism 9.0.2, **** $p<0.0001$, *** $p<0.001$, ** $p<0.01$, n.s. - not significant).

Supplementary Table S1, related to STAR Methods (Cryo-EM Sample Preparation, Data Collection and Processing; Model Building and Refinement; Quantification and Statistical Analysis). Data table for cryo-EM data collection, refinement and validation statistics of *(S)*-SG-094-bound *Hs*TPC2 and *(R)*-SG-094-bound *Hs*TPC2

	***Hs*TPC2 with *(S)*-SG-094**	***Hs*TPC2 with *(R)*-SG-094**
**Microscope**	Titan Krios	Titan Krios
**Detector**	K2	K2
**Voltage (kV)**	300	300
**Magnification**	105,000	105,000
**Collection mode**	Counting, superresolution	Counting, superresolution
**Electron exposure (e/Å ^{2})**	38	42
**Number of frames**	31	36
**Pixel size (Å)**	0.4145	0.4145
**Defocus range (µm; steps)**	-0.8 to -2.4 (0.2)	-0.8 to -2.4 (0.2)
**Number of movies**	9,924	5,848
**Initial Number of particles**	2,508,787	2,290,202
**Number of particles after 2D classification**	751,050	198,508
**Symmetry**	C1	C1
**Number of particles used for 3D refinement**	109,417	99,232
**Map resolution (Å ; FSC threshold = 0.143)**	3.0	3.6
**Resolution range (Å)**	2.6 – 43.8	3.1 – 45.2
**Map sharpening B-factor (Å ^{2})**	80.9	94.9
**Model resolution (Å ; FSC threshold = 0.5)**	3.2	
**Non-hydrogen atoms**	10,749	
**Protein residues**	1,273	
**Ligands**	14	
**R.M.S.D**		
Bond lengths (Å)	0.002	
Bond angles (°)	0.468	
**Validation**		
Molprobity score	1.69	
Clash score	5.76	
Rotamer outliers (%)	1.53	
**Ramachandran plot**		
Favoured (%)	96.42	
Allowed (%)	3.58	
Disallowed (%)	0	
**EMDB Code**	EMD-17197	EMD-19108
**PDB Code**	8OUO	

Supplementary Table S2, related to STAR Methods (Key Resources Table). DNA sequences for synthetic codon-optimised genes

Gene	Sequence
*Hs*TPC2	ATGGCAGAACCTCAAGCAGAAAGCGAACCAGCAGCAGGTGGAGCAAGGGGTGGCGGCGGTGA<br>TTGGCCTGCTGGTTTGACCACTTACCGCAGCATCCAAGTCGGCCCTGGTGCCGCGGCCAGGTGG<br>GACCTCTGCATTGATCAGGCTGTGGTCTTCATCGAAGATGCTATTCAGTACCGCTCCATCAACC<br>ACCGGGTGGATGCCAGCTCGATGTGGCTTTACCGACGGTATTACTCGAACGTATGCCAACGGA<br>CTTTGAGCTTCACCATCTTCTTGATCCTGTTTTTGGCTTTTATCGAGACCCCATCCTCACTCACC<br>AGCACGGCGGACGTGCGCTACCGCGCTGCCCCCTGGGAGCCGCCCTGCGGCCTGACCGAGAGT<br>GTCGAGGTGCTCTGCCTGCTGGTCTTTGCGGCCGACCTCTCTGTGAAGGGTTACCTGTTCGGGT<br>GGGCCCATTTCCAGAAAAACCTTTGGCTGCTGGGCTACCTCGTGGTGCTGGTGGTGTCTCTGGT<br>GGACTGGACCGTGTCCCTGAGTCTCGTGTGTCATGAGCCCCTGCGGATCCGCCGGCTTCTCCGT<br>CCCTTCTTCCTGCTGCAGAACTCCTCTATGATGAAGAAGACCTTGAAATGCATCCGCTGGTCGC<br>TGCCGGAAATGGCCAGCGTCGGGCTGCTGCTGGCCATCCACCTGTGCCTCTTCACCATGTTCGG<br>AATGCTGCTGTTCGCTGGTGGGAAGCAGGATGATGGGCAGGACAGGGAGAGGCTGACCTACTT<br>CCAGAACCTGCCTGAGTCTCTGACTTCCCTCCTGGTGCTGCTGACCACGGCCAACAACCCCGAT<br>GTGATGATTCCTGCGTATTCCAAGAACCGGGCCTATGCCATCTTCTTCATAGTCTTCACTGTGAT<br>AGGAAGCCTGTTTCTGATGAACCTGCTGACAGCCATCATCTACAGTCAGTTCCGGGGCTACCTG<br>ATGAAATCTCTCCAGACCTCGCTGTTTCGGAGGCGGCTGGGAACCCGGGCTGCCTTTGAAGTCC<br>TATCCTCCATGGTGGGGGAGGGAGGAGCCTTCCCTCAGGCAGTTGGGGTGAAGCCCCAGAACT<br>TGCTGCAGGTGCTTCAGAAGGTCCAGCTGGACAGCTCCCACAAACAGGCCATGATGGAGAAGG<br>TGCGTTCCTACGGCAGTGTTCTGCTGTCAGCTGAGGAGTTTCAGAAGCTCTTCAACGAGCTTGA<br>CAGAAGTGTGGTTAAAGAGCACCCGCCGAGGCCCGAGTACCAGTCTCCGTTTCTGCAGAGCGC<br>CCAGTTCCTCTTCGGCCACTACTACTTTGACTACCTGGGGAACCTCATCGCCCTGGCAAACCTG<br>GTGTCCATTTGCGTGTTCCTGGTGCTGGATGCAGATGTGCTGCCTGCTGAGCGTGATGACTTCA<br>TCCTGGGGATTCTCAACTGCGTCTTCATTGTGTACTACCTGTTGGAGATGCTGCTCAAGGTCTTT<br>GCCCTGGGCCTGCGAGGGTACCTGTCCTACCCCAGCAACGTGTTTGACGGGCTCCTCACCGTTG<br>TCCTGCTGGTTTTGGAGATCTCAACTCTGGCTGTGTACCGATTGCCACACCCAGGCTGGAGGCC<br>GGAGATGGTGGGCCTGCTGTCGCTGTGGGACATGACCCGCATGCTGAACATGCTCATCGTGTTC<br>CGCTTCCTGCGTATCATCCCCAGCATGAAGCCGATGGCCGTGGTGGCCAGTACCGTCCTGGGCC<br>TGGTGCAGAACATGCGTGCTTTTGGCGGGATCCTGGTGGTGGTCTACTACGTATTTGCCATCAT<br>TGGGATCAACTTGTTTAGAGGCGTCATTGTGGCTCTTCCTGGAAACAGCAGCCTGGCCCCTGCC<br>AATGGCTCGGCGCCCTGTGGGAGCTTCGAGCAGCTGGAGTACTGGGCCAACAACTTCGATGAC<br>TTTGCGGCTGCCCTGGTCACTCTGTGGAACTTGATGGTGGTGAACAACTGGCAGGTGTTTCTGG<br>ATGCATATCGGCGCTACTCAGGCCCGTGGTCCAAGATCTATTTTGTATTGTGGTGGCTGGTGTC<br>GTCTGTCATCTGGGTCAACCTGTTTCTGGCCCTGATTCTGGAGAACTTCCTTCACAAGTGGGAC<br>CCCCGCAGCCACCTGCAGCCCCTTGCTGGGACCCCAGAGGCCACCTACCAGATGACTGTGGAG<br>CTCCTGTTCAGGGATATTCTGGAGGAGCCCGAGGAGGATGAGCTCACAGAGAGGCTGAGCCAG<br>CACCCGCACCTGTGGCTGTGCAGGtga
*Hs*TPC2^{Y436A}	ATGGCAGAACCTCAAGCAGAAAGCGAACCAGCAGCAGGTGGAGCAAGGGGTGGCGGCGGTGA<br>TTGGCCTGCTGGTTTGACCACTTACCGCAGCATCCAAGTCGGCCCTGGTGCCGCGGCCAGGTGG<br>GACCTCTGCATTGATCAGGCTGTGGTCTTCATCGAAGATGCTATTCAGTACCGCTCCATCAACC<br>ACCGGGTGGATGCCAGCTCGATGTGGCTTTACCGACGGTATTACTCGAACGTATGCCAACGGA<br>CTTTGAGCTTCACCATCTTCTTGATCCTGTTTTTGGCTTTTATCGAGACCCCATCCTCACTCACC<br>AGCACGGCGGACGTGCGCTACCGCGCTGCCCCCTGGGAGCCGCCCTGCGGCCTGACCGAGAGT

	GTCGAGGTGCTCTGCCTGCTGGTCTTTGCGGCCGACCTCTCTGTGAAGGGTTACCTGTTCGGGT<br>GGGCCCATTTCCAGAAAAACCTTTGGCTGCTGGGCTACCTCGTGGTGCTGGTGGTGTCTCTGGT<br>GGACTGGACCGTGTCCCTGAGTCTCGTGTGTCATGAGCCCCTGCGGATCCGCCGGCTTCTCCGT<br>CCCTTCTTCCTGCTGCAGAACTCCTCTATGATGAAGAAGACCTTGAAATGCATCCGCTGGTCGC<br>TGCCGGAAATGGCCAGCGTCGGGCTGCTGCTGGCCATCCACCTGTGCCTCTTCACCATGTTCGG<br>AATGCTGCTGTTCGCTGGTGGGAAGCAGGATGATGGGCAGGACAGGGAGAGGCTGACCTACTT<br>CCAGAACCTGCCTGAGTCTCTGACTTCCCTCCTGGTGCTGCTGACCACGGCCAACAACCCCGAT<br>GTGATGATTCCTGCGTATTCCAAGAACCGGGCCTATGCCATCTTCTTCATAGTCTTCACTGTGAT<br>AGGAAGCCTGTTTCTGATGAACCTGCTGACAGCCATCATCTACAGTCAGTTCCGGGGCTACCTG<br>ATGAAATCTCTCCAGACCTCGCTGTTTCGGAGGCGGCTGGGAACCCGGGCTGCCTTTGAAGTCC<br>TATCCTCCATGGTGGGGGAGGGAGGAGCCTTCCCTCAGGCAGTTGGGGTGAAGCCCCAGAACT<br>TGCTGCAGGTGCTTCAGAAGGTCCAGCTGGACAGCTCCCACAAACAGGCCATGATGGAGAAGG<br>TGCGTTCCTACGGCAGTGTTCTGCTGTCAGCTGAGGAGTTTCAGAAGCTCTTCAACGAGCTTGA<br>CAGAAGTGTGGTTAAAGAGCACCCGCCGAGGCCCGAGTACCAGTCTCCGTTTCTGCAGAGCGC<br>CCAGTTCCTCTTCGGCCACTACTACTTTGACGCCCTGGGGAACCTCATCGCCCTGGCAAACCTG<br>GTGTCCATTTGCGTGTTCCTGGTGCTGGATGCAGATGTGCTGCCTGCTGAGCGTGATGACTTCA<br>TCCTGGGGATTCTCAACTGCGTCTTCATTGTGTACTACCTGTTGGAGATGCTGCTCAAGGTCTTT<br>GCCCTGGGCCTGCGAGGGTACCTGTCCTACCCCAGCAACGTGTTTGACGGGCTCCTCACCGTTG<br>TCCTGCTGGTTTTGGAGATCTCAACTCTGGCTGTGTACCGATTGCCACACCCAGGCTGGAGGCC<br>GGAGATGGTGGGCCTGCTGTCGCTGTGGGACATGACCCGCATGCTGAACATGCTCATCGTGTTC<br>CGCTTCCTGCGTATCATCCCCAGCATGAAGCCGATGGCCGTGGTGGCCAGTACCGTCCTGGGCC<br>TGGTGCAGAACATGCGTGCTTTTGGCGGGATCCTGGTGGTGGTCTACTACGTATTTGCCATCAT<br>TGGGATCAACTTGTTTAGAGGCGTCATTGTGGCTCTTCCTGGAAACAGCAGCCTGGCCCCTGCC<br>AATGGCTCGGCGCCCTGTGGGAGCTTCGAGCAGCTGGAGTACTGGGCCAACAACTTCGATGAC<br>TTTGCGGCTGCCCTGGTCACTCTGTGGAACTTGATGGTGGTGAACAACTGGCAGGTGTTTCTGG<br>ATGCATATCGGCGCTACTCAGGCCCGTGGTCCAAGATCTATTTTGTATTGTGGTGGCTGGTGTC<br>GTCTGTCATCTGGGTCAACCTGTTTCTGGCCCTGATTCTGGAGAACTTCCTTCACAAGTGGGAC<br>CCCCGCAGCCACCTGCAGCCCCTTGCTGGGACCCCAGAGGCCACCTACCAGATGACTGTGGAG<br>CTCCTGTTCAGGGATATTCTGGAGGAGCCCGAGGAGGATGAGCTCACAGAGAGGCTGAGCCAG<br>CACCCGCACCTGTGGCTGTGCAGGtga
*Hs*TPC2^{F555E}	ATGGCAGAACCTCAAGCAGAAAGCGAACCAGCAGCAGGTGGAGCAAGGGGTGGCGGCGGTGA<br>TTGGCCTGCTGGTTTGACCACTTACCGCAGCATCCAAGTCGGCCCTGGTGCCGCGGCCAGGTGG<br>GACCTCTGCATTGATCAGGCTGTGGTCTTCATCGAAGATGCTATTCAGTACCGCTCCATCAACC<br>ACCGGGTGGATGCCAGCTCGATGTGGCTTTACCGACGGTATTACTCGAACGTATGCCAACGGA<br>CTTTGAGCTTCACCATCTTCTTGATCCTGTTTTTGGCTTTTATCGAGACCCCATCCTCACTCACC<br>AGCACGGCGGACGTGCGCTACCGCGCTGCCCCCTGGGAGCCGCCCTGCGGCCTGACCGAGAGT<br>GTCGAGGTGCTCTGCCTGCTGGTCTTTGCGGCCGACCTCTCTGTGAAGGGTTACCTGTTCGGGT<br>GGGCCCATTTCCAGAAAAACCTTTGGCTGCTGGGCTACCTCGTGGTGCTGGTGGTGTCTCTGGT<br>GGACTGGACCGTGTCCCTGAGTCTCGTGTGTCATGAGCCCCTGCGGATCCGCCGGCTTCTCCGT<br>CCCTTCTTCCTGCTGCAGAACTCCTCTATGATGAAGAAGACCTTGAAATGCATCCGCTGGTCGC<br>TGCCGGAAATGGCCAGCGTCGGGCTGCTGCTGGCCATCCACCTGTGCCTCTTCACCATGTTCGG<br>AATGCTGCTGTTCGCTGGTGGGAAGCAGGATGATGGGCAGGACAGGGAGAGGCTGACCTACTT<br>CCAGAACCTGCCTGAGTCTCTGACTTCCCTCCTGGTGCTGCTGACCACGGCCAACAACCCCGAT<br>GTGATGATTCCTGCGTATTCCAAGAACCGGGCCTATGCCATCTTCTTCATAGTCTTCACTGTGAT<br>AGGAAGCCTGTTTCTGATGAACCTGCTGACAGCCATCATCTACAGTCAGTTCCGGGGCTACCTG<br>ATGAAATCTCTCCAGACCTCGCTGTTTCGGAGGCGGCTGGGAACCCGGGCTGCCTTTGAAGTCC

	TATCCTCCATGGTGGGGGAGGGAGGAGCCTTCCCTCAGGCAGTTGGGGTGAAGCCCCAGAACT<br>TGCTGCAGGTGCTTCAGAAGGTCCAGCTGGACAGCTCCCACAAACAGGCCATGATGGAGAAGG<br>TGCGTTCCTACGGCAGTGTTCTGCTGTCAGCTGAGGAGTTTCAGAAGCTCTTCAACGAGCTTGA<br>CAGAAGTGTGGTTAAAGAGCACCCGCCGAGGCCCGAGTACCAGTCTCCGTTTCTGCAGAGCGC<br>CCAGTTCCTCTTCGGCCACTACTACTTTGACTACCTGGGGAACCTCATCGCCCTGGCAAACCTG<br>GTGTCCATTTGCGTGTTCCTGGTGCTGGATGCAGATGTGCTGCCTGCTGAGCGTGATGACTTCA<br>TCCTGGGGATTCTCAACTGCGTCTTCATTGTGTACTACCTGTTGGAGATGCTGCTCAAGGTCTTT<br>GCCCTGGGCCTGCGAGGGTACCTGTCCTACCCCAGCAACGTGTTTGACGGGCTCCTCACCGTTG<br>TCCTGCTGGTTTTGGAGATCTCAACTCTGGCTGTGTACCGATTGCCACACCCAGGCTGGAGGCC<br>GGAGATGGTGGGCCTGCTGTCGCTGTGGGACATGACCCGCATGCTGAACATGCTCATCGTGTTC<br>CGCGCCCTGCGTATCATCCCCAGCATGAAGCCGATGGCCGTGGTGGCCAGTACCGTCCTGGGC<br>CTGGTGCAGAACATGCGTGCTTTTGGCGGGATCCTGGTGGTGGTCTACTACGTATTTGCCATCA<br>TTGGGATCAACTTGTTTAGAGGCGTCATTGTGGCTCTTCCTGGAAACAGCAGCCTGGCCCCTGC<br>CAATGGCTCGGCGCCCTGTGGGAGCTTCGAGCAGCTGGAGTACTGGGCCAACAACTTCGATGA<br>CTTTGCGGCTGCCCTGGTCACTCTGTGGAACTTGATGGTGGTGAACAACTGGCAGGTGTTTCTG<br>GATGCATATCGGCGCTACTCAGGCCCGTGGTCCAAGATCTATTTTGTATTGTGGTGGCTGGTGT<br>CGTCTGTCATCTGGGTCAACCTGTTTCTGGCCCTGATTCTGGAGAACTTCCTTCACAAGTGGGA<br>CCCCCGCAGCCACCTGCAGCCCCTTGCTGGGACCCCAGAGGCCACCTACCAGATGACTGTGGA<br>GCTCCTGTTCAGGGATATTCTGGAGGAGCCCGAGGAGGATGAGCTCACAGAGAGGCTGAGCCA<br>GCACCCGCACCTGTGGCTGTGCAGGtga
