# Supplementary material for: Structural basis for inhibition of the lysosomal two-pore channel TPC2 by a small molecule antagonist
Source: Structure. 2024 Aug 8;32(8):1137–1149.e4. doi: 10.1016/j.str.2024.05.005 (PMC11511679; doi:10.1016/j.str.2024.05.005)
Supplement: Data S1. Analytical data for validation of SG-094 compound synthesis, related to STAR Methods [file mmc2.pdf]

Supplementary Data 1. Analytical data for validation of SG-094 compound synthesis, related to STAR Methods

(±)-6-Methoxy-2-methyl-7-phenoxy-1-(4-phenoxybenzyl)-1,2,3,4-tetrahydroisoquinoline – SG-094 (racemate)

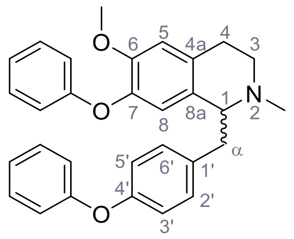

SG-094

SG-094 was synthesized according to [1], yielding the racemic mixture as colorless oil. $\mathbf{R_f}$ = 0.61 (9:1 CH_2Cl_2/MeOH). **^{1}H NMR (400 MHz, CD_3OD)** δ/ppm = 7.29 – 7.18 (m, 4H, Ph), 7.07 – 7.00 (m, 3H, 2'-H, 6'-H, Ph), 6.96 – 6.92 (m, 1H, Ph), 6.86 – 6.82 (m, 3H, 5-H, Ph), 6.79 – 6.75 (m, 2H, 3'-H, 5'-H), 6.72 – 6.68 (m, 2H, Ph), 6.10 (s, 1H, 8-H), 3.75 (dd, J = 8.7, 4.4 Hz, 1H, 1-H), 3.72 (s, 3H, OCH_3), 3.28 – 3.14 (m, 2H, α-H, 3-H), 2.96 (ddd, J = 15.5, 9.1, 6.6 Hz, 1H, 4-H), 2.83 – 2.74 (m, 3H, α-H, 3-H, 4-H), 2.53 (s, 3H, NCH_3). **^{13}C NMR (101 MHz, CD_3OD)** δ/ppm = 159.8 (qPh), 159.0 (qPh), 156.9 (C-4'), 151.7 (C-6), 143.0 (C-7), 135.5 (C-1'), 132.2 (C-2', C-6'), 131.9 (C-4a or C-8a), 130.8 (Ph), 130.6 (C-4a or C-8a), 130.4 (Ph), 124.0 (Ph), 123.0 (Ph), 122.6 (C-8), 119.9 (C-3', C-5'), 119.5 (Ph), 117.3 (Ph), 114.1 (C-5), 65.7 (C-1), 56.3 (OCH_3), 47.3 (C-3), 42.6 (NCH_3), 39.9 (C-α), 26.6 (C-4). **IR (ATR)** $\tilde{\nu}_{max}$/cm^{-1} = 3038, 2937, 2838, 2793, 1588, 1505, 1487, 1218, 749, 690. **HRMS (ESI):** calcd. for $C_{30}H_{30}NO_3$ $(M+H)^+$ 452.22202; found 452.22163. **Purity (HPLC):** > 96% (λ = 210 nm).

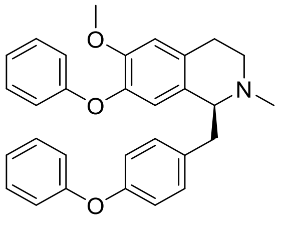

(S)-SG-094

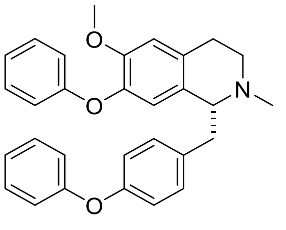

(R)-SG-094

Separation of racemic SG-094 *via* chiral HPLC (9.5:0.5 *n*-heptane/isopropanol + 0.45% diethylamine) yielded the two enantiomers (S)-SG-094 (colorless oil, retention time: ~ 8 min) and (R)-SG-094**,** (colorless oil, retention time: ~ 11 min). (S)-SG-094**:** $[\boldsymbol{\alpha}]_{\boldsymbol{D}}^{\mathbf{22}}$ **=** + 74.5 (c = 0.043, $CHCl_3$). (R)-SG-094**:** $[\boldsymbol{\alpha}]_{\boldsymbol{D}}^{\mathbf{22}}$ **=** - 52.5 (c = 0.057, $CHCl_3$).

[1] Müller et al. (2021) Gene editing and synthetically accessible inhibitors reveal role for TPC2 in HCC cell proliferation and tumor growth. *Cell Chem. Biol.* **28**, 1119–1131, doi: https://doi.org/10.1016/j.chembiol.2021.01.023
