# Supplementary material for: Structural basis for inhibition of the lysosomal two-pore channel TPC2 by a small molecule antagonist
Source: Structure. 2024 Aug 8;32(8):1137–1149.e4. doi: 10.1016/j.str.2024.05.005 (PMC11511679; doi:10.1016/j.str.2024.05.005)
Supplement: Document S2. Article plus supplemental information [file mmc3.pdf]

Structure

Article

Structural basis for inhibition of the lysosomal two-pore channel TPC2 by a small molecule antagonist

Graphical abstract

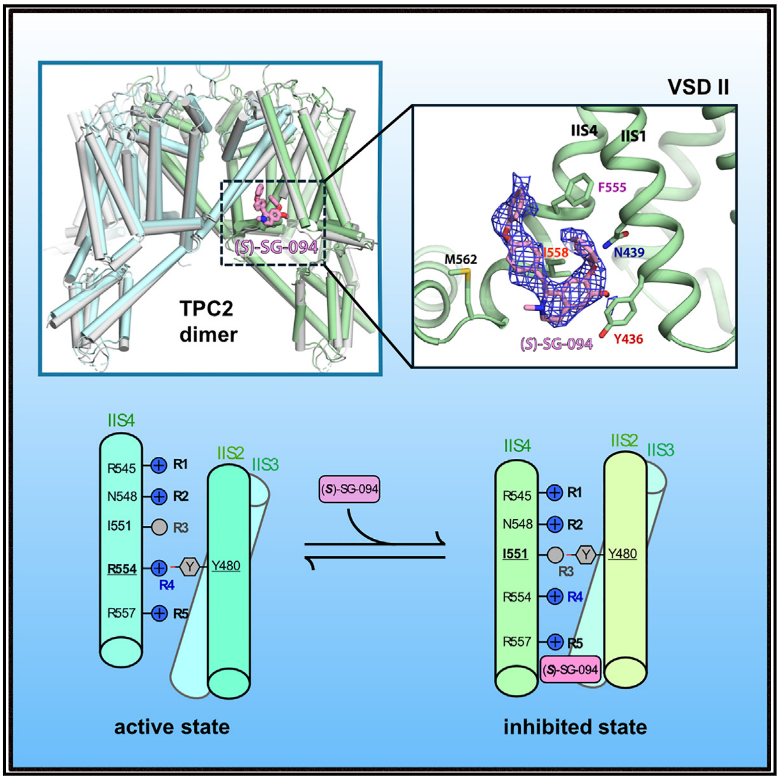

Authors

Gamma Chi, Dawid Jaślan,
Veronika Kudrina, ..., Franz Bracher,
Christian Grimm, Katharina L. Dürr

Correspondence

gamma.chi@cmd.ox.ac.uk

In brief

Two pore channels are lysosomal cation channels, and inhibition of the two-pore channel 2 (TPC2) has emerged as a potential therapeutic strategy for the treatment of cancers and viral infections. Here, Chi et al. demonstrate that its antagonist SG-094 induces asymmetrical structural changes to TPC2, stabilizing it in an inactive state.

Highlights

- Synthetic TPC2 antagonist *(S)*-SG-094 binds to human TPC2's VSD II domain
- *(S)*-SG-094 inhibits *Hs*TPC2 by stabilizing VSD II in an induced inactive state
- *Hs*TPC2 has two well-coordinated lipid-binding sites in VSD I domain

Chi et al., 2024, Structure *32*, 1137–1149
August 8, 2024 © 2024 The Authors. Published by Elsevier Inc.
https://doi.org/10.1016/j.str.2024.05.005

CellPress

Structure

CellPress
OPEN ACCESS

Article

Structural basis for inhibition of the lysosomal two-pore channel TPC2 by a small molecule antagonist

Gamma Chi,[1,2,11,12,*] Dawid Jaślan,[3,11] Veronika Kudrina,[3,11] Julia Böck,[3,11] Huanyu Li,[1,2] Ashley C.W. Pike,[1,2] Susanne Rautenberg,[4,6] Einar Krogsaeter,[3,7] Tina Bohstedt,[1,2] Dong Wang,[1,2] Gavin McKinley,[1,2] Alejandra Fernandez-Cid,[1,2,8] Shubhashish M.M. Mukhopadhyay,[1,2,9] Nicola A. Burgess-Brown,[1,2,8] Marco Keller,[4] Franz Bracher,[4] Christian Grimm,[3,5] and Katharina L. Dürr[1,2,10]

[1]Centre for Medicines Discovery, Nuffield Department of Medicine, University of Oxford, Nuffield Department of Medicine Research Building, Oxford OX3 7FZ, UK

[2]Structural Genomics Consortium, Nuffield Department of Medicine, University of Oxford, Nuffield Department of Medicine Research Building, Oxford OX3 7FZ, UK

[3]Walther-Straub-Institut für Pharmakologie und Toxikologie, Medizinische Fakultät, Ludwig-Maximilians-Universität, Nussbaumstrasse 26, 80336 Munich, Germany

[4]Department of Pharmacy, Center for Drug Research, Ludwig-Maximilians-Universität, Butenandtstrasse 7, 81377 Munich, Germany

[5]Immunology, Infection and Pandemic Research IIP, Fraunhofer Institute for Translational Medicine and Pharmacology ITMP, Munich/Frankfurt, Germany

[6]Present address: Eurofins BioPharma Product Testing GmbH Munich, Robert-Koch-Straße 3a, 82152 Planegg

[7]Present address: The J. David Gladstone Institutes, 1650 Owens Street, San Francisco, CA,94158, USA

[8]Present address: Exact Sciences Ltd., The Sherard Building, Edmund Halley Road, The Oxford Science Park, Oxford, OX4 4DQ, UK

[9]Present address: Exscientia Ltd., The Schrödinger Building, Heatley Road, The Oxford Science Park, Oxford, OX4 4GE, UK

[10]Present address: OMass Therapeutics, Ltd., Building 4000, Chancellor Court, John Smith Drive, ARC Oxford, OX4 2GX, UK

[11]These authors contributed equally

[12]Lead contact

*Correspondence: gamma.chi@cmd.ox.ac.uk

https://doi.org/10.1016/j.str.2024.05.005

SUMMARY

Two pore channels are lysosomal cation channels with crucial roles in tumor angiogenesis and viral release from endosomes. Inhibition of the two-pore channel 2 (TPC2) has emerged as potential therapeutic strategy for the treatment of cancers and viral infections, including Ebola and COVID-19. Here, we demonstrate that antagonist SG-094, a synthetic analog of the Chinese alkaloid medicine tetrandrine with increased potency and reduced toxicity, induces asymmetrical structural changes leading to a single binding pocket at only one intersubunit interface within the asymmetrical dimer. Supported by functional characterization of mutants by Ca^{2+} imaging and patch clamp experiments, we identify key residues in S1 and S4 involved in compound binding to the voltage sensing domain II. SG-094 arrests IIS4 in a downward shifted state which prevents pore opening via the IIS4/S5 linker, hence resembling gating modifiers of canonical VGICs. These findings may guide the rational development of new therapeutics antagonizing TPC2 activity.

INTRODUCTION

Two-pore channel 2 (TPC2) is a nicotinic acid adenine dinucleotide phosphate (NAADP) and phosphatidylinositol-(3,5)-diphosphate ($PI(3,5)P_2$)-activated Na^+ and Ca^{2+}-permeable channel in the endolysosomal system.[1–3] TPC2 plays important roles in intracellular vesicle trafficking, autophagy, and exocytosis[4–8] and is associated with a number of human disease pathologies.[9–11] For example, TPC2-mediated calcium signaling is associated with cell proliferation,[12] angiogenesis,[10,13] and metastasis,[14,15] therefore affecting cancer progression at all stages. Loss of TPC2 results in cholesterol accumulation in liver hepatocytes and fatty liver diseases[16] while gain-of-function mutations in humans result in pigmentation defects.[17,18] More recently, it was discovered that TPC2-mediated calcium signaling is critical for Ebola virus and Coronavirus to escape from lysosomes into cytoplasm after internalization,[19–22] hence attracting further interest as subject of host-targeting antiviral therapeutics.[11,19,23] This has led to non-selective TPC2 antagonists tetrandrine (NCT04308317, Henan Provincial People's Hospital, China) and verapamil (NCT04351763, Uniwersytet Mikołaja Kopernika w Toruniu, Poland) being explored in clinical trials as potential drug candidates for the treatment of Ebola virus and Sars-Cov-2 infections.[11]

A number of synthetic ligands have been developed for TPC2,[12,13,24,25] some of them based on their natural analogs.

This is an open access article under the CC BY license (http://creativecommons.org/licenses/by/4.0/).

TPC2 can be activated by several endogenous molecules, most notably PI(3,5)P_2 and NAADP,[3,26] with recent evidence suggesting that NAADP indirectly interacts with TPC2 via LSM12 or JPT2.[27–31] Antagonist *trans*-Ned19 was identified by *in silico* screening of compound libraries for NAADP-like molecules, targeting TPC1 and TPC2.[25] Additionally, TPC2-A1-N and TPC2-A1-P are synthetic small molecule TPC2 agonists which functionally (but not structurally) mimic NAADP and PI(3,5)P_2, respectively, and they show similar ion selectivities compared to the natural ligands.[32]

Plant extracts are another major source for TPC2 antagonists, with the alkaloid tetrandrine from the liana *Stephania tetrandra* (Menispermaceae), naringenin from grapefruit (*Citrus paradisi*, Rutaceae), and related flavonoids such as pratensein (MT-8) and duartin (UM-9) from *Dalbergia parviflora* being major examples.[13,33,34] These compounds are nonselective two-pore channel inhibitors and act on other cellular systems as well, hence requiring optimization for improved TPC2 selectivity. Recently, a series of simplified synthetic analogs of tetrandrine was developed and shown to have enhanced efficiency of TPC2 inhibition in cellular assays,[12] as well as higher potency to inhibit proliferation of cancer cells in a mouse model. Importantly, these newly developed synthetic antagonists (SG-005 and SG-094) also exhibited reduced toxicity toward non-cancerous cells. The study also highlighted that these tetrandrine congeners are superior to naringenin and Ned-19 regarding their anti-tumor activity and hence represent promising candidates for further development into small molecule therapeutics to treat human cancers.

High-resolution structures of mouse TPC1 and human TPC2 (*Hs*TPC2) were recently determined with X-ray crystallography and cryo-electron microscopy (cryo-EM),[35,36] where they showed homodimeric assembly of two subunits, each consisting of twelve transmembrane helices, resembling a tandem repeat of a 6 TM helices (6 TM I in N-terminal half and 6 TM II in C-terminal half) observed in voltage-gated ion channels (VGICs) of the Kv, Nav, and Cav families. Other similarities to VGICs are the domain-swapped architecture where the S1-S4 voltage-sensing domain (VSD) of one subunit is in close contact to the S5-S6 of the pore domain (PD) of the neighboring subunit for the C-terminal VSD II, as well as the presence of an arginine-rich S4 domain, facing a so-called charge-transfer center (CTC) in S2. Although both VSDs in *Hs*TPC2 are insensitive to voltage, VSD II can be converted to an active sensor by introducing a charge-sensing arginine into R3 of IIS4 (I551 in wild-type *Hs*TPC2)[35] and the channel can become voltage-sensitive in the presence of certain tricyclic agonists.[37] For *Hs*TPC2, structures in closed and an open-like state in complex with activating ligand PI(3,5)P_2 were determined, providing significant insight to its activation mechanism.[35] PI(3,5)P_2 interacts with TPC2 at a pocket between VSD I and IS4/5 linker helix (cytoplasmic helix between S4 and S5 helices of N-terminal half), with the polar interaction between its phosphoinositides and surrounding positively charged residues K203, K204, K208, and R329 thought to be crucial for its opening. The structures suggest these interactions bring IS6 helix closer to PI(3,5)P_2, which then leads to conformational change where the central pore dilates to an open-like state.

We sought to further enable *Hs*TPC2 as a therapeutic target by structurally characterizing its antagonist-bound state. We determined the structure of *Hs*TPC2 in complex with a pure *(S)*-enantiomer of a synthetic antagonist SG-094 with cryo-EM, where we uncovered not only the binding pocket and binding mode of the compound, further backed up by site-directed mutagenesis, patch-clamp experimentation, and Ca^{2+} imaging analysis, but also its influence on TPC2 conformation. Our structural data will provide necessary information for guiding further optimization of the TPC2 antagonists for higher affinity and increased selectivity.

RESULTS

Overall structure of *Hs*TPC2 in complex with antagonist *(S)*-SG-094

To obtain purified *Hs*TPC2 for structure determination, we expressed a full-length *Hs*TPC2 with L11A/L12A mutations in the dileucine lysosomal targeting motif in HEK293F-GnTI$^-$ cells, followed by purification using a similar protocol to publication by She et al.[35] *(S)*-SG-094 compound was supplemented with final concentration of 5 μM in all buffers throughout purification in order to maximize its incubation time with *Hs*TPC2 for saturation of its binding site, followed by overnight incubation with 200 μM *(S)*-SG-094 compound after the last purification step, which would lead to closed-state *Hs*TPC2 in complex with the inhibitor. We determined the *Hs*TPC2 structure at 2.8 Å nominal resolution with cryo-EM, with most regions of the protein displaying clear side chain features and high resolutions estimated in local resolution map. We identified a sub-state of *Hs*TPC2 with significant movement of VSD II domains, and refinement of this set of particles yielded a *Hs*TPC2/SG-094 complex structure at 3.0 Å nominal resolution (Figures 1A and 1B). *Hs*TPC2/*(S)*-SG-094 complex structure has consistent pore and EF-hand domain structures to the published closed-state *Hs*TPC2 structure (PDB: 6NQ2).[35] There is a small molecule feature consistent with the chemical structure of SG-094 in the VSD II domain of one subunit in this electrostatic potential (ESP) map (Figure 1C), clearly indicating its location and binding mode. Interestingly, VSD II domain of the other subunit in the homodimer does not show such a feature (Figure 2C), which suggests an asymmetric binding of *(S)*-SG-094 to *Hs*TPC2 with 1:1 dimer-to-ligand stoichiometry instead of 1:2 which would be expected with a full occupancy (the subunit with *(S)*-SG-094 will be referred to as subunit A, and the one without inhibitor will be referred to as subunit B). This is supported by the co-operativity values of SG-094 calculated from the results by Müller et al.,[12] which is −1.3 (SD = 0.4) against agonist TPC2-A1-P and −0.9 (SD = 0.5) against TPC2-A1-N, suggesting either singular binding or no-co-operativity between the two potential sites.

We also attempted to determine the structure of *Hs*TPC2 in complex with *(R)*-SG-094 enantiomer using similar methods. This has led to a 3.5 Å reconstruction of *Hs*TPC2 in near-identical state as that with *(S)*-SG-094. While this map shows a small molecule ESP feature at the same site as the binding pocket of *(S)*-SG-094 (Figure S5B), it is not defined well enough to ascertain its binding mode, possibly due to the lower achieved resolution. Therefore, we infer that *(R)*-SG-094 binds at the same site as *(S)*-SG-094 and has a similar *Hs*TPC2 inhibition mechanism; however, we will refer only to *Hs*TPC2 structure with *(S)*-SG-094 for detailed analysis.

CellPress
OPEN ACCESS

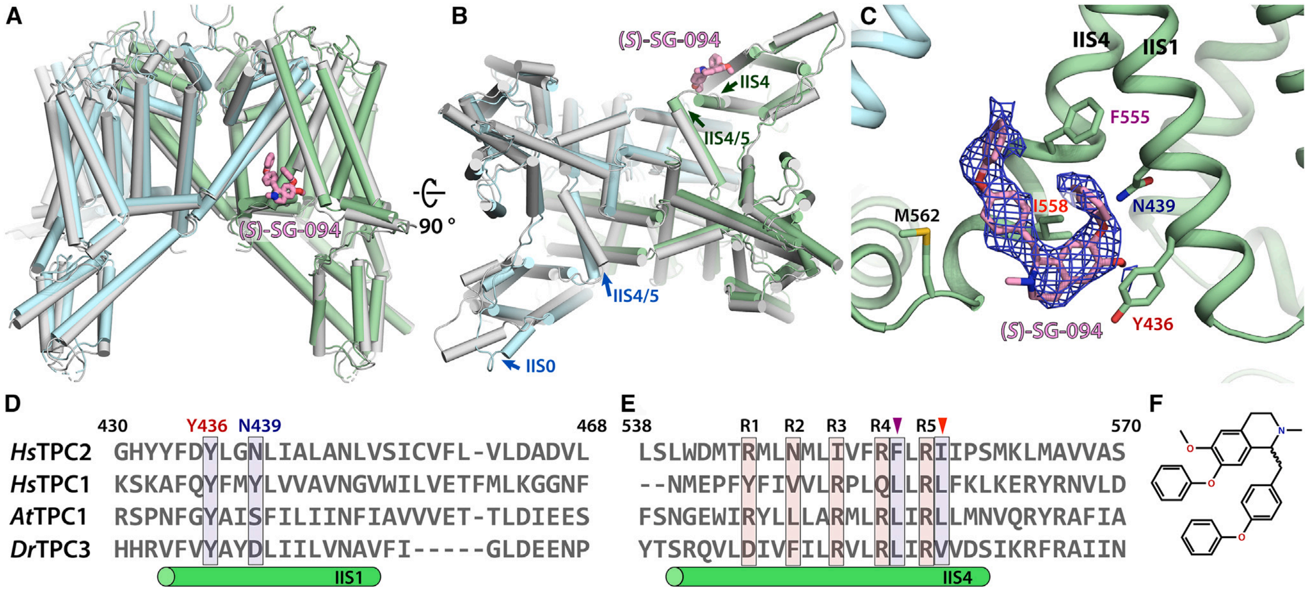

Figure 1. Structural overview of *Hs*TPC2 in complex with (S)-SG-094

(A and B) Overall structure of *Hs*TPC2 in complex with *(S)*-SG-094. Light green, Subunit A (in complex with SG-094); Light cyan, Subunit B without *(S)*-SG-094; Gray – *Hs*TPC2 in apo state (PDB: 6NQ1). For the cytoplasmic view (B), structural differences between apo and *(S)*-SG-094-bound HsTPC2 are marked with arrows.

(C) Close-up view of the *(S)*-SG-094 binding pocket located at the interface between S5 in the pore domain I of subunit B (light cyan) and S1 from voltage-sensing domain II of subunit A (light green). *(S)*-SG-094 (pink stick representation) is fitted into ESP map (blue mesh, $\sigma = 5.0$). Subunit A residues in proximity of SG-094 are shown as light green sticks.

(D and E) Sequence alignment of regions IIS1 (D) and IIS4 (E) in VSDII with other members of the TPC family. Gating charge residues R1-R5 are highlighted in light pink, and residues in the *(S)*-SG-094 binding pocket are highlighted in blue.

(F) Chemical structure of SG-094.

(S)-SG-094 stabilizes *Hs*TPC2 in an induced inactive state

(S)-SG-094 is located at a cleft between IIS1 and IIS4 helices of VSD II on the cytoplasmic side (Figure 1C) near IIS4/5 linker helix. The experimentally determined binding mode of *(S)*-SG-094 was validated with *in silico* docking experiment around this pocket (Figures 3D and 3E) which also led to similar binding modes. Of the residues involved in *(S)*-SG-094 binding, only Y436 in IIS1 is fully conserved in other members of the TPC family, whereas the remaining interactions with the compound are mediated by non-conserved residues (Figures 1D and 1E). Interestingly, this location is distinct to antagonists GX-936 and ProTx2 against Nav1.7 (Figures 3A and 3F–3H)[38] while still similar in that all three act on VSD for their channel inhibition, and is analogous to the binding sites for PI(4,5)P_2 in Kv7.4 and positive modulator LuAG00563 for Kv3.1 (Figures 3A–3C and 3I–3K),[39,40] with the latter having a similar binding mode to *(S)*-SG-094. The compound lacks a polar interaction, and it mainly forms hydrophobic interactions with nearby residues, such as Y436, F555, and I558. In particular, the pi stacking interaction of the *N*-methyl-tetrahydroisoquinoline residue of *(S)*-SG-094 with Y436 appears highly important to the compound binding.

To further validate the observed binding site by functional experiments, we performed cell-based calcium release assays of wild-type *Hs*TPC2, *Hs*TPC2^{Y436A}, *Hs*TPC2^{N439A}, and *Hs*TPC2^{F555A} (Figures 4A–4F). Whereas *Hs*TPC2^{WT} shows robust SG-094-mediated inhibition of Ca^{2+} signals in response to TPC-A1-N or TPC-A1-P (Figures 4A and 4C), *Hs*TPC2^{Y436A} is activated by both agonists regardless of the presence of SG-094 (Figures 4B and 4D), hence supporting our hypothesis that Y436 is essential for SG-094 binding. We also confirmed this interpretation by electrophysiology, measuring TPC2-mediated currents in whole-cell patch-clamp recordings of plasma-membrane-targeted *Hs*TPC2 variants (Figure 5). In line with the Ca^{2+} imaging data, we observe significant inhibition by SG-094 only for wild-type channels (Figures 5A and 5C), while *Hs*TPC2^{Y436A} shows identical currents in response to TPC2-A1-N activation in the presence and absence of antagonist SG-094 (Figures 5B and 5C). For two additional mutants in the putative *(S)*-SG-094 binding pocket, *Hs*TPC2^{N439A} and *Hs*TPC2^{F555A}, we have either lost activation almost completely (*Hs*TPC2^{N439A}) or we still observed inhibition (albeit to a slightly lesser extent and with lower significance) by the compound (*Hs*TPC2^{F555A}) in Ca^{2+} imaging experiments (Figures 4E and 4F), indicating that these two residues play a less important/less specific role for SG-094 antagonism.

To complement these experiments, we utilized a tryptophan fluorescence-based thermal shift assay as a third validating method for SG-094 binding to *Hs*TPC2 and its mutants (Figures S2A–S2C). The melting curve profile for *Hs*TPC2^{WT} shows significant differences between apo sample and SG-094-added sample (FigureS2A) such as biphasic melting profile for the latter. For both *Hs*TPC2^{Y436A} and *Hs*TPC2^{F555E}, however, there is very little difference between apo samples and SG-094-added samples (Figures S2B and S2C), indicating reduced binding of the compound on both mutants. These indicate that both hydrophobic environment of the binding pocket and the pi

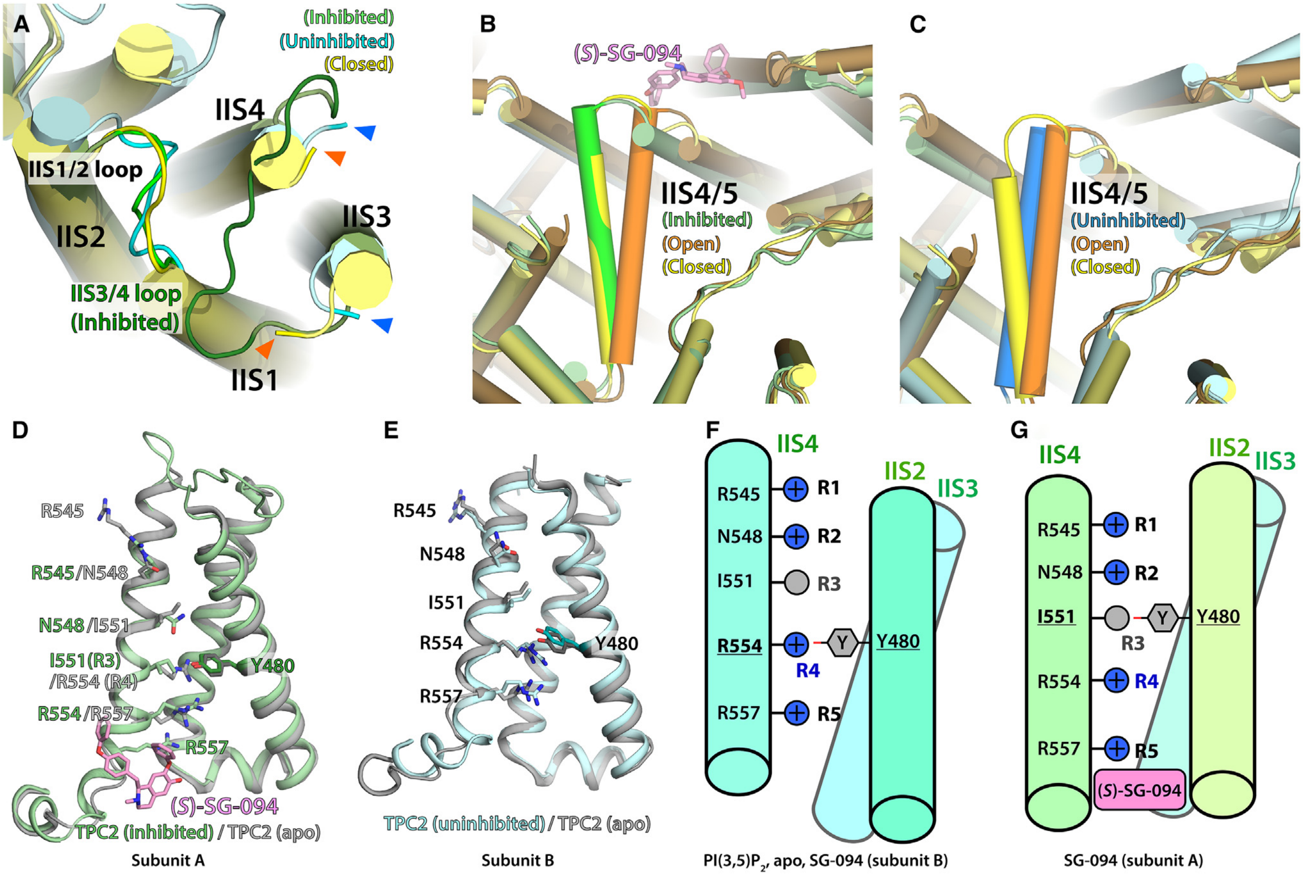

Figure 2. SG-094 traps *Hs*TPC2 in a closed state through rearrangements of IIS4 in VSD II and the IIS4/S5 linker

(A–G) Comparison of the VSD II arrangement (A and D–G) and S4/S5 linker II arrangement (B and C) of the inhibited subunit A (light green), the inhibitor-free subunit B (light cyan) in the *(S)*-SG-094-bound *Hs*TPC2 structure and the respective arrangements observed in the two identical subunits of the closed (apo, yellow, PDB: 6NQ1) or PI(3,5)P_2-bound activated structure (orange, PDB: 6NQ0).

(A) Comparison of the lumenal side of VSD of *Hs*TPC2 structures. Green, subunit A; Light cyan, subunit B; Yellow, Apo-*Hs*TPC2 (PDB: 6NQ1). For the IIS3/4 loop region, missing model due to flexibility is marked with blue (subunit B) or orange (apo state) triangles.

(B) Cytoplasmic view of subunit A (green) shows IIS4/5 in closed-like state. Yellow, Apo-*Hs*TPC2; Orange, PI(3,5)P_2-bound *Hs*TPC2 in open state (PDB: 6NQ0).

(C) Cytoplasmic view of subunit B (blue) shows IIS4/5 in open-like state.

(D) VSD II domain of subunit A (light green) shows shift of IIS4 helix with voltage-sensing residues (R545–R554) C-terminally shifting by a full turn compared to apo-*Hs*TPC2 (light gray).

(E) VSD II of subunit B (light cyan) is in the same state as apo-*Hs*TPC2 (light gray).

(F and G) Schematic diagrams for simplified views of *Hs*TPC2's VSD II in resting state including apo, PI(3,5)P_2-bound closed and PI(3,5)P_2-bound open states (F), and SG-094-inhibited state (G). IIS1 is omitted for clarity.

stacking interaction with Y436 are important in SG-094 binding to TPC2.

Our structure shows *(S)*-SG-094 inducing conformational change on *Hs*TPC2 at various levels. At the local level, *(S)*-SG-094 triggers several side chains to reposition (Figure 6A), creating a binding pocket (Figure 6C) which is absent in the 2-fold symmetrical apo state structure (Figure 6D). Such shift not only results in a better fit of *(S)*-SG-094 but also makes the pocket more hydrophobic as the ε-amino group of K563 and the hydroxyl group of Y432 are moved away from the pocket (Figures 6A, 6C, and 6D). Interestingly, this shift effectively results in the replacement of K536's amine group with the tertiary amine of *(S)*-SG-094 at near-identical position (Figure 6A). Hence, the electronic repulsion between K563 and *(S)*-SG-094's amine group may be a key process involved in the local conformational change. Another notable local reorganization is the movement of M562 and K563 in opposite directions to their open state positions (Figure 6A). These local changes induce reorganization of the two adjacent extracellular loops S1/S2 and S3/S4 (Figures 6B and 2A) and also have profound consequences on the conformation of the IIS4/5 linker helix at the immediate downstream (Figures 2B and 2C) which will be discussed in the next section.

(S)-SG-094 leads to a significant conformational shift on the overall structure of the VSD II domain, directly affecting its IIS4 position (Figure 2). *Hs*TPC2 has a voltage sensor-like, arginine-rich motif (R545/N548/I551/R554/R557) on its IIS4 helix facing toward residue Y480 on IIS2 helix (Figures 2D–2G). These correspond to gating charges R1-R5 and a conserved Phe in the hydrophobic charge transfer center in canonical VGICs,

CellPress
OPEN ACCESS

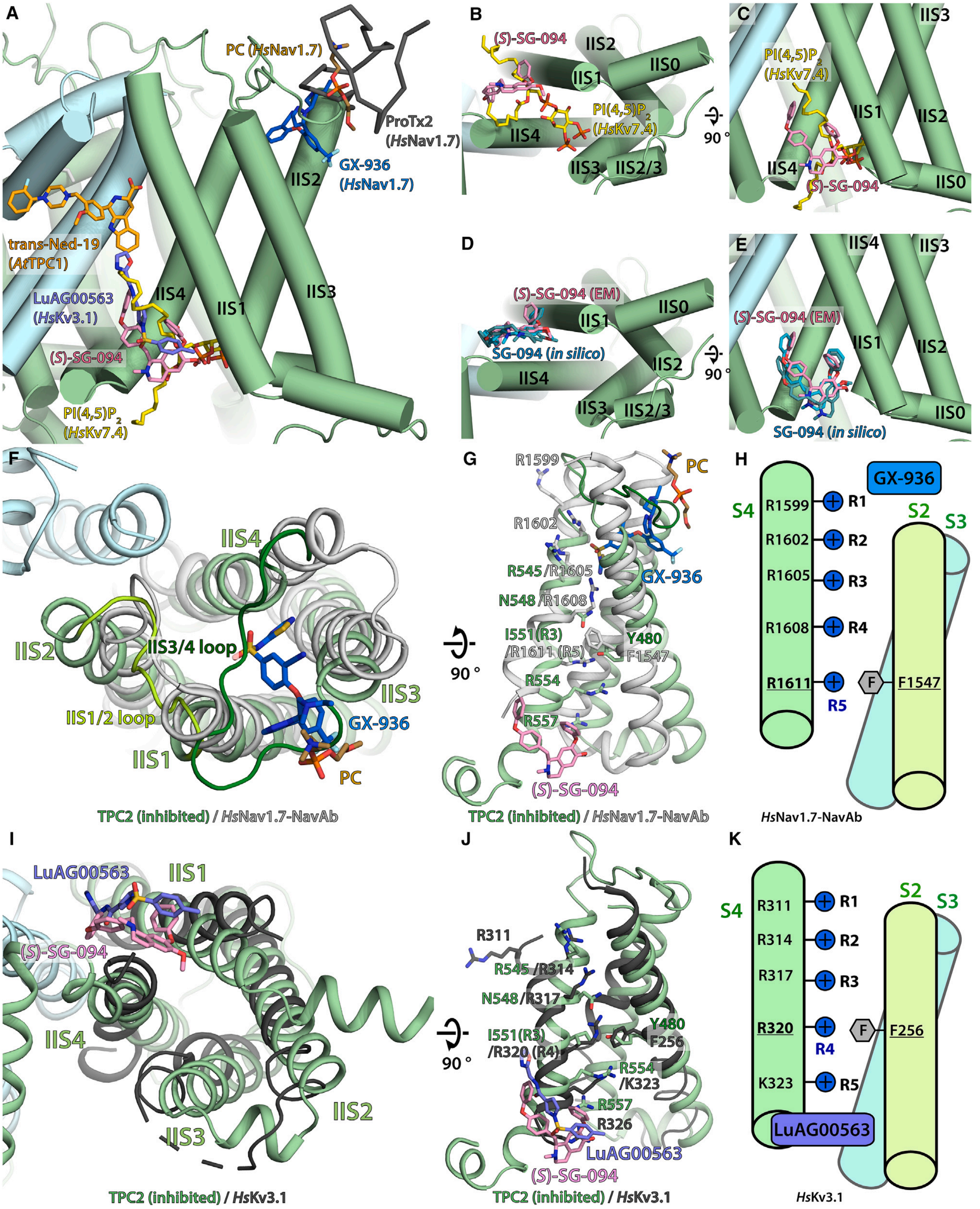

(legend on next page)

respectively. Y480 is in contact with R554 (R4) in apo-closed state of *Hs*TPC2, which is the case with uninhibited subunit B as well (Figures 2E and 2F). On the other hand, SG-094 binding to VSD II induces a shift of IIS4 helix toward the cytoplasmic (i.e., C-terminal) side by one full turn, which results in Y480 interacting with I551 (R3) instead (Figure 2G). This shifts VSD II's voltage sensors from an intermediate-closed state to a resting-closed state (following the scheme proposed by Kintzer et al.)[36] in a similar configuration to *At*TPC1^{D454N}.[41]

Asymmetric SG-094 binding leads to structural changes in both subunits

(S)-SG-094's influence on VSD II has long-range effect on *Hs*TPC2 conformation. Notably, the IIS3/4 linker loop, which is too flexible to feature in apo state and PI(3,5)P_2-bound *Hs*TPC2 maps, appears in the *(S)*-SG-094-complexed samples (Figure 2A). Given *(S)*-SG-094's direct effect on IIS4 helix, this is likely due to the stabilization of IIS4 helix in the closed state. The IIS3/4 loop forms hydrophilic interactions with the backbone peptides IIS1/2 loop (Figure 6B). This is in contrast to TPC1 structures where IIS3/4 loop is not only shorter but also too distant from IIS1/2 loop (>9 Å) for interactions.[42] These loops are critical for the function of TPCs, as evidenced by two out of three residues critical for calcium-sensing in *At*TPC1 (D454 and E528) being located near IIS1/2 and IIS3/4, respectively.[36] S3/4 loop in VSD is important for the function of other ion channels such as Shaker, Kv3.1, and Cav1.1 channels as well.[43–45] Additionally, this position of IIS3/4 in TPC2 is analogous to the location of GX-936 antagonist in *Hs*Nav1.7/NavAb chimera structure,[38] where the compound stabilizes the VSD in active-like state (Figures 3H and6B). Therefore, stabilization of IIS3/4 loop by SG-094 may further contribute to TPC2 inhibition by interfering with activation mediated by this region.

(S)-SG-094-induced structural changes to VSD II have wider implications on the overall conformation of *Hs*TPC2. The cytoplasmic shift of IIS4 helix in subunit A and associated movement of residues M562 and K563 lead to the stabilization of II S4/5 linker helix in a closed state with an extra helical turn on its N-terminal side (Figures 2B and 7J). Locking of IIS4/5 helix in closed state will contribute to inhibition of PI(3,5)P_2-mediated opening of *Hs*TPC2, as the movement of II S4/5 helix toward VSD II was put forward as a key step in the opening process in the previously published *Hs*TPC2 structures. Indeed, in our thermal shift assays, SG-094 reverses PI(3,5)P_2-induced increase in *Hs*TPC2 stability to near apo state level (Figure S2) supporting this hypothesis.

Interestingly, SG-094-induced structural changes to VSD II in subunit A are coupled to several minor changes in the VSD II of uninhibited subunit B leading to the collapse of the latter's inhibitor-binding pocket. Structural alignment of apo-TPC2 to SG-094-bound subunit A suggests that this is a hinge-like long-range effect of the ligand binding (Figures 1B and 7), given that the two subunits are symmetrical in published TPC structures.[35,42] Additional helical turn and rotation of subunit A's IIS4/5 helix caused by a downward movement of IIS4 helix would have shifted subunit B by less than 1 Å on its inhibitor-facing side. This would then amplify to 2–3 Å in EF hand and VSD I, which would lead to rotation of IIS0 helix (Figure 7G), and then to nearly 8 Å in VSD II on the N-terminal side of the inhibitor-binding pocket (Figure 1B). This would also cause rotation of subunit B's IIS4/5 helix in an opposite direction to an open-like state on the C-terminal side of the pocket (Figures 2C and 7G). The resultant rotation of VSD II and IIS4/5 helix toward each other in subunit B would have completely closed the inhibitor-binding pocket, preventing its access (Figure 6E). Therefore, it can be postulated that the binding of *(S)*-SG-094 in one subunit leads to the closure of its binding pocket in the other subunit, explaining the asymmetric binding of the compound to *Hs*TPC2 homodimer.

SG-094's mode of HsTPC2 inhibition may be different from that of tetrandrine

Our cell-based experiments indicate that tetrandrine may have a different mode of *Hs*TPC2 inhibition to SG-094 (Figures 5 andS6). Whereas SG-094 fails to inhibit agonist-induced current for *Hs*TPC2^{Y436A} mutant (Figure 5C), tetrandrine can still inhibit the current regardless of this mutation (Figure S6D). This suggests that tetrandrine's binding mode does not involve Y436, which is a key residue for SG-094 binding. Given that the proximity of *(S)*-SG-094 to Y436 and the generally exposed environment of the antagonist-binding pocket in our structure, it is unlikely for tetrandrine to bind to *Hs*TPC2 at the same site without Y436's involvement.

Tetrandrine and SG-094 have different inhibition responses to activators TPC2-A1-N and TPC2-A1-P (Figures 5 andS6), providing further evidence for their different inhibition mechanisms. Whereas 10 μM SG-094 is sufficient for nearly full inhibition of HsTPC2 activated by TPC2-A1-N (Figure 5C), even 50 μM SG-094 achieves only partial inhibition of HsTPC2 activated by

Figure 3. Comparison of the SG-094 binding site in *Hs*TPC2 to other small molecule modulators targeting VSDs in TPC, Nav, and Kv channels
(A) Antagonist *trans*-Ned-19 in *At*TPC1 (PDB: 5DQQ) binds on the other side (extracellular) of VSD II/pore domain interface compared to *(S)*-SG-094. Agonists PI(4,5)P2 in *Hs*Kv7.4 (PDB: 8BYL) and LuAG00563 in *Hs*Kv3.1 (PDB: 7PQU) bind at the same site as *(S)*-SG-094. Inhibitors GX-936 for *Hs*Nav1.7 (PDB: 5EK0) and ProTx2 for *Hs*Nav1.7 (PDB: 6N4R) bind at S2/S3 pocket on the extracellular side.
(B and C) *(S)*-SG-094 binds at the same site at VSD II as PI(4,5)P_2 does at the VSD of Kv7.4.
(D and E) Experimentally determined *(S)*-SG-094 model (pink) matches closely with *in silico* docked models of *(S)*-SG-094 (teal, cyan).
(F) Extracellular view onto VSDII in the *Hs*Nav1.7/NavAb chimera (PDB: 5EK0), illustrating binding of antagonist GX-936 to the cleft between IIS1 and IIS3.
(G) Side view of VSDII of *Hs*Nav1.7/NavAb in complex with GX-936 superposed with VSDII from the *Hs*TPC2/*(S)*-SG-094 complex structure.
(H) Cartoon schematic of VSDII of *Hs*Nav1.7/NavAb in complex with GX-936, illustrating the positioning of voltage-sensing residues R1-R5 (in IIS4) with respect to F1547 of the CTC in IIS2. In contrast to SG-094 which stabilizes IIS4 of TPC2 in a downward-shifted state, GX-936 arrests Nav1.7 in a fully activated (upward shifted) state of IIS4, which leads to Nav1.7 antagonism via inactivation.
(I) Cytoplasmic view of VSD of *Hs*Kv3.1 in complex with LuAG00563.
(J) Side view of VSD of HsKv3.1 in complex with LuAG00563.
(K) Cartoon schematic of VSD of HsKv3.1 in complex with LuAG00563, illustrating the positioning of voltage-sensing residues R1-R5 with respect to F256. S4 is in upward shifted state, in line with LuAG00563's positive modulatory effect on *Hs*Kv3.1.

CellPress
OPEN ACCESS

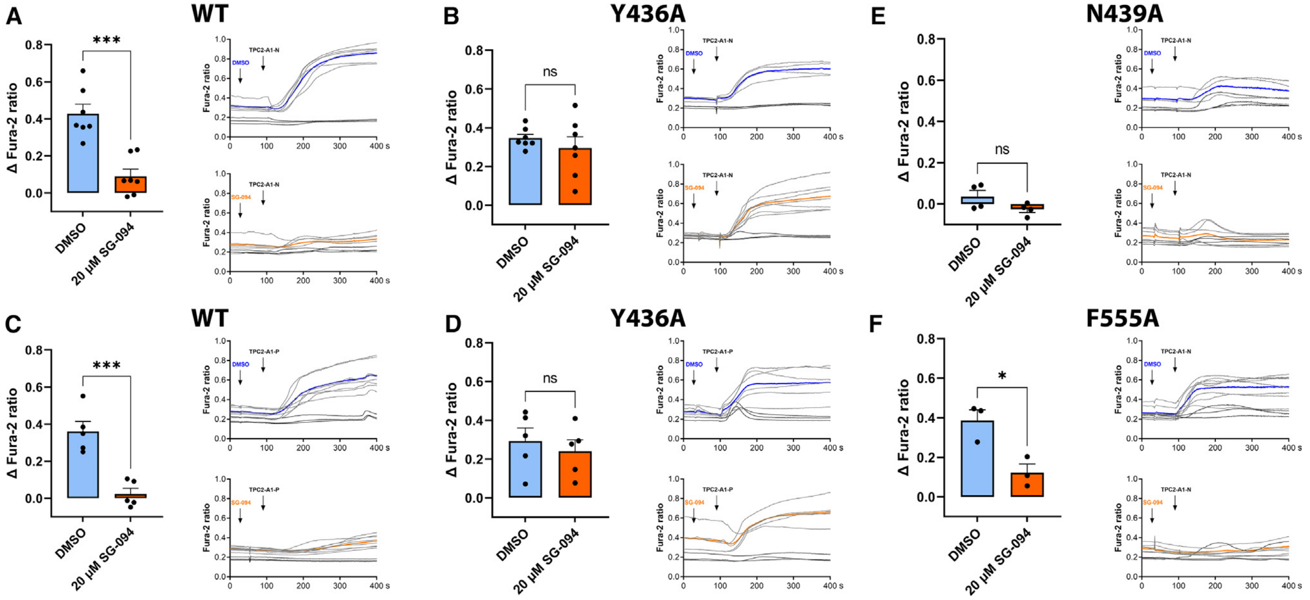

Figure 4. Decreased *Hs*TPC2 inhibition by SG-094 for binding site mutations Y436A, N439A, and F555A in Ca^{2+} imaging experiments

(A–F) The curve graphs are representative Ca^{2+} signals recorded from HEK293 cells transiently transfected with plasma-membrane *Hs*TPC2$^{L11A/L12A}$-eYFP variants. The cells were loaded with the ratiometric Ca^{2+} indicator Fura-2 and stimulated with either 10 μM TPC2-A1-N (A, B, E, and F) or 30 μM TPC2-A1-P (C and D). Blue curves represent control measurements (mean values), where 0.1% DMSO was applied instead of SG-094 before stimulation, orange curves demonstrate the effects caused by the addition of 20 μM SG-094 (mean values). Transfected single-cell traces are shown in light gray, untransfected cell traces are shown in dark gray. Experiments were performed at least in triplicates and statistical analyses of the maximal changes in the Fura-2 ratio (mean ± SEM, unpaired t test using GraphPad Prism 9.0.2, ***$p < 0.001$) are shown within the bar charts.

TPC2-A1-P (Figure S6B). Among a list of several possible explanations, one is that SG-094 is directly inhibiting the activation mechanism of TPC2-A1-N and not directly doing so for TPC2-A1-P. On the other hand, tetrandrine shows similar levels of inhibition for HsTPC2 activated by TPC2-A1-N and TPC2-A1-P (Figures S6D and S6F), suggesting a mode of inhibition that is different to SG-094's.

As we were not successful with our attempts to determine the *Hs*TPC2/tetrandrine complex structure and tetrandrine does not induce shift in *Hs*TPC2 thermostability in our nanoDSF experiment, it is difficult to fully explain such differences between the two structurally similar antagonists on their effects on *Hs*TPC2. However, observations from our cell-based experiments suggest that SG-094 and tetrandrine may be different classes of *Hs*TPC2 inhibitors despite their chemical similarities.

TPC2 has two well-coordinated phospholipid binding sites per subunit

Structures of two-pore channels often have a number of lipid-like molecules featuring around their transmembrane surfaces, as evidenced by the modeling of hydrophobic tails in *At*TPC1.[36] We also observed a number of lipid/detergent-like features in the detergent belt, however, we did not model them as it was not certain if they were phospholipid tails or if they were hydrophobic moieties of glyco-diosgenin (GDN) detergent. Notable exceptions are two features around VSD I of each subunit (Figure 8A), which clearly show both hydrophobic tails and polar head groups consistent with phospholipids. One of them, PL1, is located at a cytoplasmic cleft formed by IS3, IS4, and IS4/5 helices (Figure 8B), the same site as where PI(3,5)P_2 binds (Figures 8E and 8F). The binding mode of its head group is similar

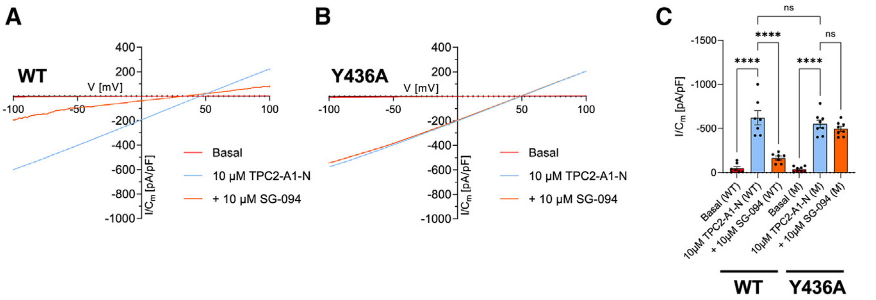

Figure 5. Loss of TPC2 current inhibition by SG-094 for binding site mutant Y436A in whole-cell patch-clamp recordings

Representative current density-voltage (I/Cm-V) relation of transiently expressed, plasma-membrane-targeted *Hs*TPC2$^{L11A/L12A}$-eYFP variants, WT (A) and Y436A mutant (B). Channels were activated by application of TPC2-A1-N (10 μM, blue traces), followed by application of the TPC antagonist SG-094 (10 μM, orange traces). Statistical analysis of experiments is shown in (C), with each dot representing mean of 5–10 technical replicate measurements (mean ± SEM; n = 7–8 independent experiments; one-way ANOVA, Tukey's post hoc test using GraphPad Prism 9.0.2, ****$p < 0.0001$, n.s. - not significant).

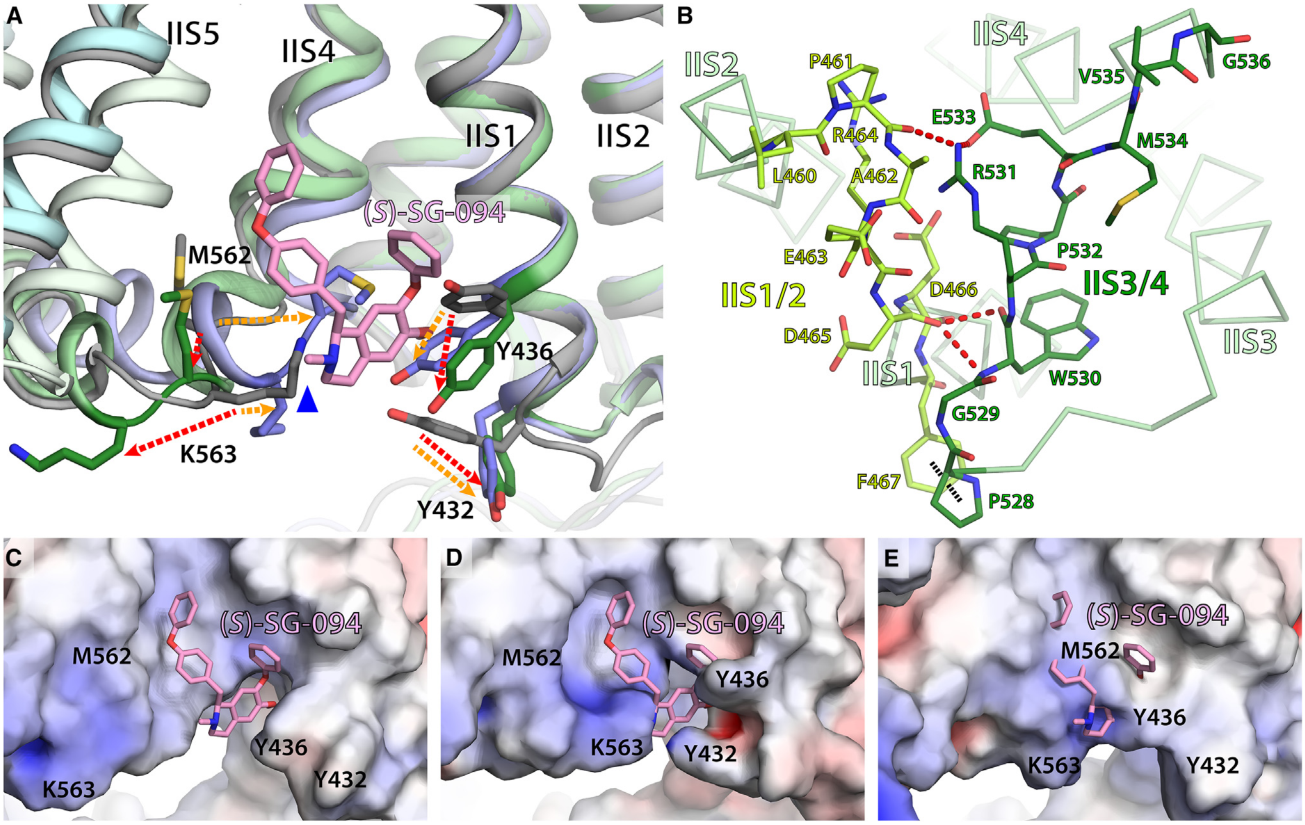

Figure 6. SG-094 binding causes asymmetrical reorganization of VSD II resulting in a single antagonist binding cleft within the *Hs*TPC2 dimer

(A) Several residues (Y432, Y436, M562, and K563) in S4 and S1 of the VSD II of both subunits reposition in response to *(S)*-SG-094 binding to *Hs*TPC2. Gray, apo-TPC2 (PDB: 6NQ1); Green, SG-094-bound subunit A; Light blue, subunit B without *(S)*-SG-094. Red arrows, residue displacement from apo state to subunit A of *(S)*-SG-094-bound state; orange arrows, residue displacement from apo state to subunit B of *(S)*-SG-094-bound state. Electrostatic/steric clash between K563's amine group and *(S)*-SG-094's tertiary amine is marked with blue triangle.

(B) Interaction between II S1/2 (light green) and II S3/4 (dark green) loops on the extracellular side of VSD II in subunit A.

(C–E) APBS-generated electrostatic surface representations (−5.0 to 5.0 kT/e) of *(S)*-SG-094-binding sites. (C) In subunit A, the binding pocket is open to accommodate *(S)*-SG-094, and the surface is generally electroneutral. (D) In apo-TPC2, Y432 and K563 would sterically clash with hypothetical *(S)*-SG-094, and the environment is more hydrophilic than *(S)*-SG-094-bound state. (E) In subunit B, the binding site has fully closed due to the movement of M562 and K563, making it sterically difficult for *(S)*-SG-094 to bind.

to PI(3,5)P_2 and forms polar interactions with W157 in IS3 as well as K207 and R210 in IS4/5. Compared to the published structure with PI(3,5)P_2, one of PL1's fatty acid tails extends much further toward the pore domain into a hydrophobic groove bounded by I S4/5, I S5, II S5, and II S6 (Figure 8C). Since this cleft is where a number of ion channel modulators bind, it is possible for some TPC2 ligands to target this site as well, either as open-state pore blockers or as PI(3,5)P_2 lipid analogs. This is indeed where tricyclic antidepressants are hypothesized to bind,[24] although this hypothesis remains to be experimentally confirmed.

PL2 is also located at a cytoplasmic cleft between VSD I and pore domain formed by IS1, IS4, and IIS5, on the other side of IS4-IIS5 axis compared to PL1 (Figure 8B). PL2's hydrophobic tails extend toward a hydrophobic pocket formed by IS1, IS4, IIS5, and IIP2 helices (Figure 8D), with sharp turn of one tail around F98 seen with both subunits. Its head group forms a number of well-coordinated polar interactions with N198, S199, S200, M201, and R580, indicating high affinity for phospholipids at this site. While none of these residues are directly involved in PI(3,5)P_2 binding, it is still possible for phospholipid in this pocket to play a role by stabilizing the VSD I and pore domain interfaces given its proximity. Interestingly, this is a potential binding site for a PI(3,5)P_2-analogous agonist TPC2-A1-P,[24] providing support to the significance of this lipid-binding site in TPC2 modulation.

DISCUSSION

Our structural investigation shows that SG-094 inhibits *Hs*TPC2 via interactions with its VSD II domain, which we have subsequently validated with both cell-based and protein-based assays. Some of the residues surrounding *(S)*-SG-094 binding pocket are poorly conserved (Figures 1D and 1E), such as N439 (Y449 in MmTPC1, S437 in *At*TPC1, and D425 in drTPC3), F555 (arginine in other TPCs), and M562 (K551 in MmTPC1, V548 in *At*TPC1, and I529 in *Dr*TPC3). As SG-094 has been shown to inhibit both TPC1 and TPC2,[12] such sequence variation is not expected to have a major influence on the compound selectivity. However, this sequence diversity still leaves the potential to

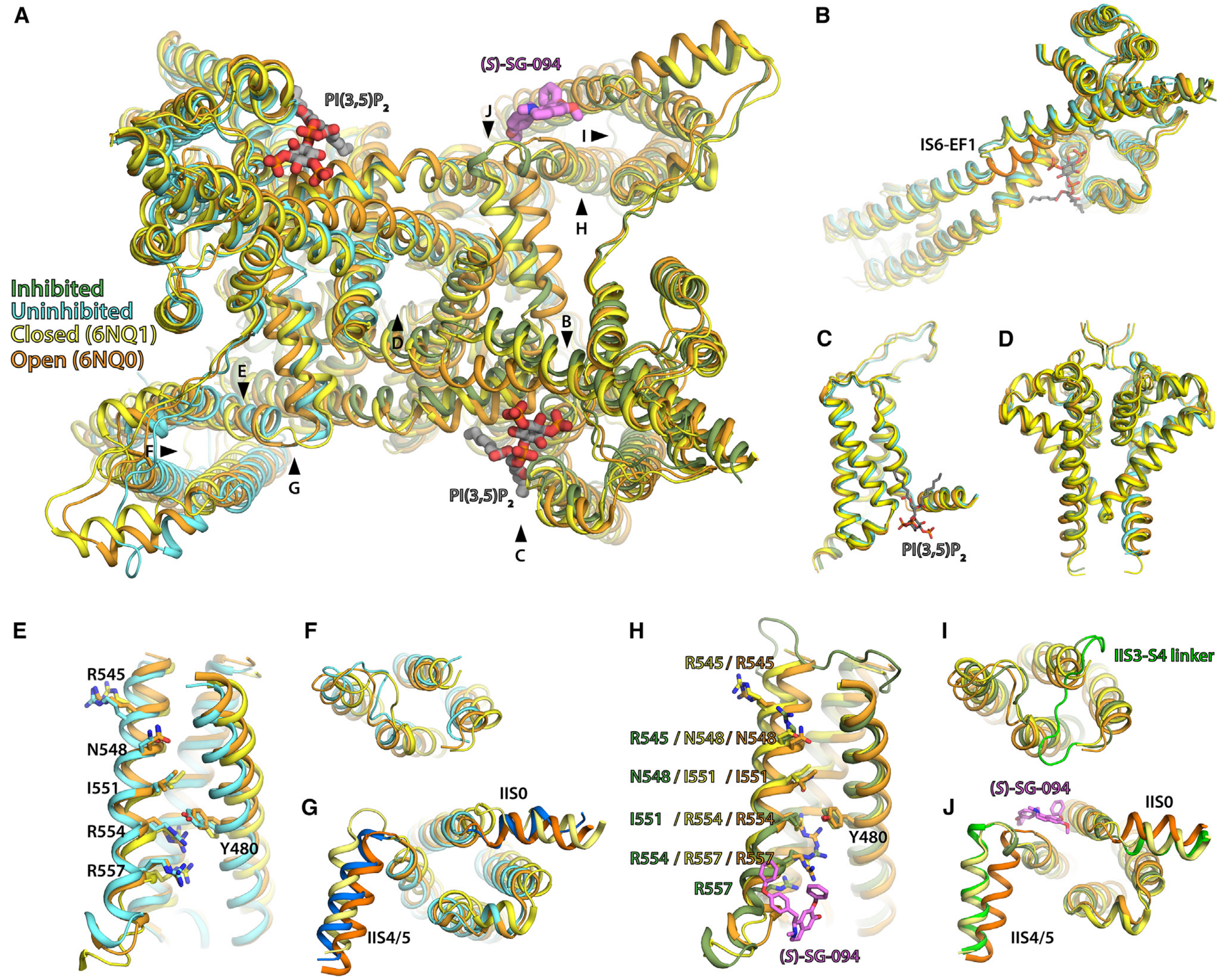

Figure 7. Comparison of *(S)*-SG-094-bound HsTPC2 to open and closed state structures of HsTPC2

(A) Overall structural comparison between the inhibitor-bound HsTPC2 (subunit A: green, subunit B: cyan), closed state *Hs*TPC2 (PDB: 6NQ0; yellow), and open state *Hs*TPC2 (PDB: 6NQ1; orange). Key sites of interest highlighted in the next panels are marked with arrows and labels.

(B) Close-up comparison of the IS6 and EF hand domain of HsTPC2 structures. Only open state TPC2 has continuous helix from IS6 to EF1 in response to PI(3,5) P_2 binding, with the other three being similar.

(C) Close-up comparison of VSD I domain. All four compared structures have the same conformation.

(D) Close-up comparison of pore region. All three compared structures have similar conformations, with only the open state having slightly dilated IS6 toward the cytoplasmic side.

(E) Close-up views of VSD for uninhibited subunit B against open and closed states. Voltage sensor residues for all three structures are in same positions.

(F) Close-up views of the lumenal side of VSD for subunit B against open and closed states. All three have similar structures.

(G) Close-up views of the cytoplasmic side of VSD for subunit B against open and closed states. IIS0 for subunit B is shorter than the other two models and has slightly rotated away from the protein center (blue). IIS4/5 helix of subunit B (blue) is more aligned with IIS4/5 of open state model (darker orange) than the closed one (light yellow).

(H) Close-up views of VSD for *(S)*-SG-094-bound subunit A against open and closed states. Voltage sensor residues for subunit A has shifted downward by one turn.

(I) Close-up views of the lumenal side of VSD for subunit A against open and closed states. IIS3/4 linker of subunit A (bright green) is ordered whereas the models for the other two structures are missing due to flexibility.

(J) Close-up views of the cytoplasmic side of VSD for subunit A against open and closed states. IIS0 for all three structures are in similar states. IIS4/5 helix of subunit A aligns well with the closed state model, and has an extra turn on the N-terminal side.

further optimize SG-094 as a compound selective to either TPC1 or TPC2.

Our data proposes SG-094 to inhibit *Hs*TPC2 function by stabilizing it in a closed state as opposed to blocking an open pore. Our structure of *(S)*-SG-094-bound *Hs*TPC2 shows the inhibitor-bound subunit in a closed conformation as evidenced by its VSD II and IIS4/5 helix. In addition, our thermal shift assay shows SG-094 reversing PI(3,5)P_2's influence on *Hs*TPC2, hence preventing

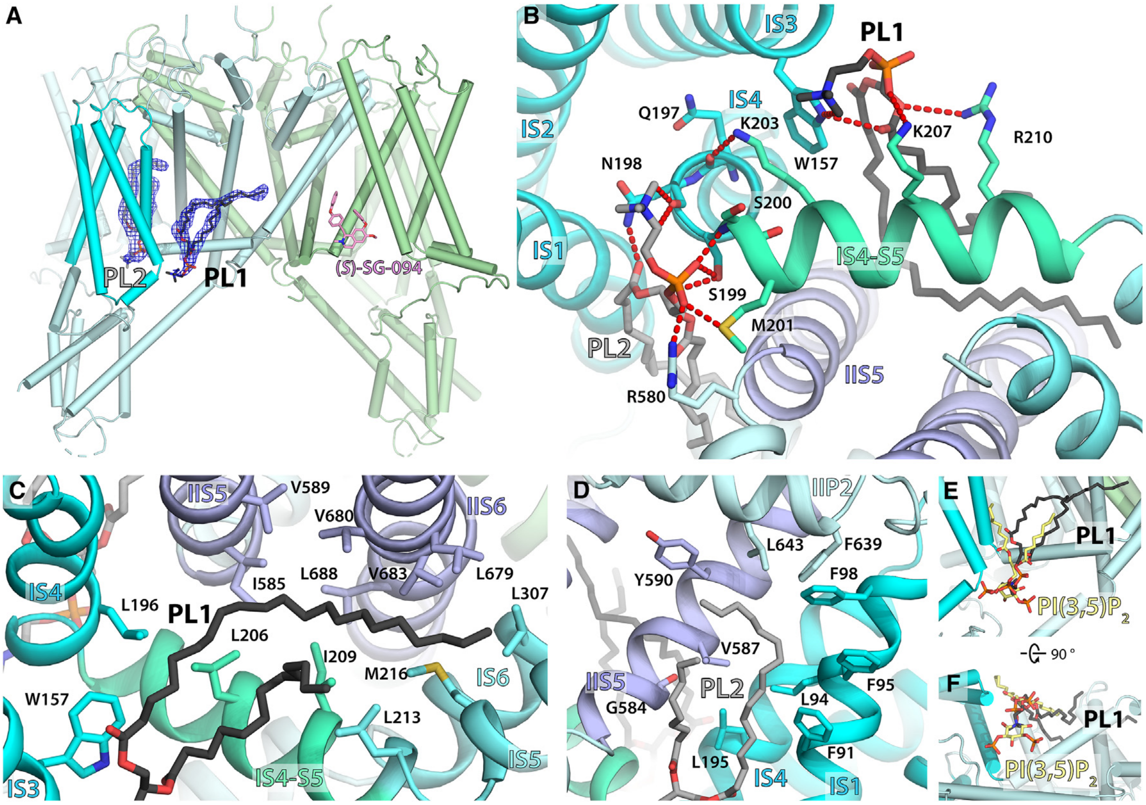

Figure 8. SG-094-bound *Hs*TPC2 reveals a second lipid binding site, distinct from PI(3,5)P_2 (agonist) binding site

(A) Overall structure of *Hs*TPC2 denoting the locations of the two phospholipids (PL1, black; PL2, gray). Light green, *(S)*-SG-094-bound subunit A; Light cyan, subunit B without SG-094; Cyan, VSD I domain.

(B) Cytoplasmic view of the interface between VSD I and pore domain shows the head groups of PL1 and PL2 forming hydrophilic interactions with *Hs*TPC2 residues.

(C) PL1's hydrophobic tail extends to a hydrophobic pocket formed by I S4/5, I S5, and II S5.

(D) PL2's hydrophobic tail extends to a hydrophobic region bounded by I S1, I S4, and II S5.

(E and F) Overlaying PI(3,5)P_2 in an open *Hs*TPC2 structure (PDB: 6NQ0) shows it with a similar binding mode to PL1.

it from transitioning to an open state. A hypothetical open state blocker should not have had much effect on apo-TPC2 in closed state or should have provided only mild stabilization, and it should have provided additional effect on PI(3,5)P_2-spiked *Hs*TPC2, however, this is not the case in our experiment. Instead, the negation of thermostability by one molecule against the other suggests that TPC2 may have been destabilized by continuous allosteric competition for its conformational state by two opposing forces exerted by the molecules. Although PI(3,5)P_2 does not appear to directly influence VSD2 in an open state *Hs*TPC2 structure,[35] electrophysiological recordings indicate that it likely leads to conformational transition of VSD2 in membrane environment,[46] supporting this hypothesis.

Our structure shows *(S)*-SG-094 stabilizing *Hs*TPC2 in closed state via multiple potential mechanisms. *(S)*-SG-094's primary effect is on VSD II, which plays an important role in voltage sensing for transition between closed and open states as evidenced by structural studies of *At*TPC1.[47] *(S)*-SG-094 stabilizes VSD II in a closed-like state where I551 (third voltage-sensing residue, or R3) is in contact with Y480 instead of R554 (R4) observed for the apo state. Interestingly, a similar downward-shifted S4 conformation was suggested to explain the voltage-inhibited phenotype of the I551R variant of *Hs*TPC2 which requires positive voltages as second stimulus for activation,[35] while wild-type *Hs*TPC2 can be activated across the full voltage range solely by PI(3,5)P_2, in line with the more activated S4 status seen in the apo and PI(3,5)P_2-activated structures.

SG-094 may sterically prevent TPC2 from turning to an open state by keeping IIS4/5 helix from rotating toward VSD II. In this mechanism, SG-094 induces residues M562 and K563 to move in opposite direction to their open state positions, which in turn affects both IIS4 helix in VSD II (pulled toward cytoplasmic side) and II S4/5 helix in the pore domain (locked into closed state). This has a follow-on effect of extending II S4/5 helix by nearly a full turn on its N-terminal side. Coincidentally, L564P polymorphism associated with the M484L effect on hair color is located at this loop-helix transition zone.[17] Therefore, it will be interesting to compare affinity to SG-094 between the two variants, 564L and 564P, with potential implications on the efficacy of SG-094-derived drugs. TPC2 inhibition via VSD II domain is an interesting discovery on its own which warrants further studies. While VSD II's role in TPC1 especially in relation to calcium and voltage sensing is well established,[47] its functional significance in TPC2 is much less known. For example, there is little conformational change

observed for VSD II with PI(3,5)P_2-bound state,[35] where IIS4/5 helix could shift to an open state independent of VSD II's voltage sensors. However, studies of TPC2 polymorphism among human population show VSD II to be a key domain in its gating, with several sequence variations implicated in phenotypes such as hair color (L/P564 and M/L484), height, BMI, and bone mineral density (L564P), or type-2 diabetes (M484L).[17,18,48] In this context, it is not surprising that a ligand such as SG-094 would act on this domain for TPC2 modulation, and it may even be used as a chemical probe specific to VSD II domain. Intriguingly, a number of well-studied toxins and small molecule antagonists for voltage-gated sodium channels (Navs) are also targeting VSDs,[38,49,50] albeit at a different location, hence highlighting unforeseen parallels in the mechanism of inhibition. Interestingly, the binding site of SG-094 is highly analogous to that of a positive Kv3.1 modulator LuAG00563,[40] with even their binding modes being very similar (Figures 3A, 3I, and 3J). This suggests that a ligand targeting this site can modulate VSD in either positive or negative direction depending on its interactions. Additionally, such similarity shows that the binding site and mode of SG-094 is, while novel for an inhibitor, not unprecedented in the context of VSD-mediated channel modulation.

Presence of two endogenous phospholipids in VSD I domain are the other major ligands in our TPC2 structure (detailed in supplementary results). The proximity of the two phospholipids as well as interacting residues suggest potential co-operativity, which could be an interesting area of study to better understand PI(3,5)P_2-mediated TPC2 activation. With both lipids, one of their hydrophobic tails extends into deep hydrophobic pockets of the pore domain where its inhibitors are thought to bind.[51] Therefore, it will be interesting to study if TPC2 inhibitors act by disrupting the protein-lipid interactions and to develop TPC2 modulators targeting such sites.

Conclusion

Lysosomal dysfunction emerged as a key process in the pathogenesis of a number of diseases such as cancer, neurodegenerative diseases, metabolic diseases, and more recently, viral infections. TPC2's profile as one of the primary cation channels in lysosomes has made it a prime drug target, with both activating and inhibiting ligands potentially having therapeutic potentials. Our investigation shows a small molecule compound SG-094 to inhibit TPC2 by stabilizing its VSD II and IIS4/5 helix in closed state. This enhances our understanding of TPC2 function by revealing VSD II as an important domain for its gating and forms the structural basis for a lead optimization program based on SG-094 as a TPC2-selective antagonist. Modeling of two phospholipids at VSD I with hydrophobic tails extending to the potential drug-binding pocket will also provide insight into the roles of lipids in ion channel function and provide valuable input for designing modulators targeting these sites.

STAR★METHODS

Detailed methods are provided in the online version of this paper and include the following:

- KEY RESOURCES TABLE
- RESOURCE AVAILABILITY
  - Lead contact
  - Materials availability
  - Data and code availability
- EXPERIMENTAL MODEL AND STUDY PARTICIPANT DETAILS
- METHOD DETAILS
  - Molecular biology, virus production and protein expression
  - SG-094 compound synthesis and preparation
  - Protein purification
  - nanoDSF thermal shift assay
  - Cryo-EM sample preparation, data collection and processing
  - Model building and refinement
  - Compound docking simulation
  - Site-directed mutagenesis, transformation and isolation of plasmid DNA
  - Cell culture, transient transfection, Ca^{2+} imaging experiments and whole cell patch-clamp electrophysiology
- QUANTIFICATION AND STATISTICAL ANALYSIS

SUPPLEMENTAL INFORMATION

Supplemental information can be found online at https://doi.org/10.1016/j.str.2024.05.005.

ACKNOWLEDGMENTS

We thank Elizabeth Maclean, Dr. Loic Carrique, Dr. Helen Duyvesteyn and Dr. Thomas Walters at Oxford Particle Imaging Center (Oxford, UK) for their assistance with electron microscopes. We also thank Dr. Brian Marsden at Center for Medicines Discovery (Oxford, UK) for his assistance with cluster maintenance. This research was carried out with funding from the Innovative Medicines Initiative 2 Joint Undertaking (JU) under grants agreements no. 875510 (EUbOPEN) and no. 115766 (UltraDD). The JU receives support from the European Union's Horizon 2020 research and innovation program and EFPIA and Ontario Institute for Cancer Research, Royal Institution for the Advancement of Learning, McGill University, Kungliga Tekniska Hoegskolan, and Diamond Light Source. This research was funded in whole, or in part, by the Wellcome Trust (grant no. 106169/Z/14/Z). For the purpose of Open Access, the author has applied a CC BY public copyright license to any Author Accepted Manuscript version arising from this submission. This research was also funded by the German Research Foundation (grant no. SFB/TRR152 P04, SFB1328 A21, DFG GR4315/2-2, DFG GR4315/4-1, and BR 1034/7-1), and we would like to thank Alzheimer's Research UK for their support and funding for the ARUK-Oxford Drug Discovery Institute (grant no. ARUK-2021DDI-OX). Oxford Particle Imaging Center was funded by a Wellcome Trust JIF award (grant no. 060208/Z/00/Z) and is supported by equipment grants from WT (093305/Z/10/Z).

AUTHOR CONTRIBUTIONS

H.L., T.B., A.F.-C., N.B.B., and D.W. performed molecular biology. G.C., H.L., T.B., G.M., and S.M.M.M. performed protein expression. G.C. and H.L. performed protein purification. G.C. carried out cryo-EM sample preparation, data collection, and data processing. G.C. and A.C.W.P. refined the structure model. G.C. performed nanoDSF thermal shift assay. G.C. performed *in silico* docking and binding free energy calculations. V.K. performed Ca^{2+} imaging experiments, D.J. and J.B. carried out whole-cell patch-clamp experiments, S.R., M.K., and F.B. contributed chemical compounds used in this study. G.C. and E.K. performed virtual mutant analysis (docking). G.C. and K.L.D. prepared the manuscript. G.C., K.L.D., F.B., M.K., and C.G. revised the manuscript.

DECLARATION OF INTERESTS

The authors declare no competing interests.

Received: May 18, 2023
Revised: October 22, 2023
Accepted: May 3, 2024
Published: May 29, 2024

REFERENCES

1. Pitt, S.J., Reilly-O'Donnell, B., and Sitsapesan, R. (2016). Exploring the biophysical evidence that mammalian two-pore channels are NAADP-activated calcium-permeable channels. J. Physiol. *594*, 4171–4179. https://doi.org/10.1113/jp270936.
2. Grimm, C., Hassan, S., Wahl-Schott, C., and Biel, M. (2012). Role of TRPML and two-pore channels in endolysosomal cation homeostasis. J. Pharmacol. Exp. Ther. *342*, 236–244. https://doi.org/10.1124/jpet.112.192880.
3. Pitt, S.J., Funnell, T.M., Sitsapesan, M., Venturi, E., Rietdorf, K., Ruas, M., Ganesan, A., Gosain, R., Churchill, G.C., Zhu, M.X., et al. (2010). TPC2 is a novel NAADP-sensitive Ca2+ release channel, operating as a dual sensor of luminal pH and Ca2+. J. Biol. Chem. *285*, 35039–35046. https://doi.org/10.1074/jbc.M110.156927.
4. García-Rúa, V., Feijóo-Bandín, S., Rodríguez-Penas, D., Mosquera-Leal, A., Abu-Assi, E., Beiras, A., María Seoane, L., Lear, P., Parrington, J., Portolés, M., et al. (2016). Endolysosomal two-pore channels regulate autophagy in cardiomyocytes. J. Physiol. *594*, 3061–3077. https://doi.org/10.1113/jp271332.
5. Lin, P.H., Duann, P., Komazaki, S., Park, K.H., Li, H., Sun, M., Sermersheim, M., Gumpper, K., Parrington, J., Galione, A., et al. (2015). Lysosomal two-pore channel subtype 2 (TPC2) regulates skeletal muscle autophagic signaling. J. Biol. Chem. *290*, 3377–3389. https://doi.org/10.1074/jbc.M114.608471.
6. Lu, Y., Hao, B., Graeff, R., and Yue, J. (2013). NAADP/TPC2/Ca(2+) Signaling Inhibits Autophagy. Commun. Integr. Biol. *6*, e27595. https://doi.org/10.4161/cib.27595.
7. Pereira, G.J.S., Hirata, H., Fimia, G.M., do Carmo, L.G., Bincoletto, C., Han, S.W., Stilhano, R.S., Ureshino, R.P., Bloor-Young, D., Churchill, G., et al. (2011). Nicotinic acid adenine dinucleotide phosphate (NAADP) regulates autophagy in cultured astrocytes. J. Biol. Chem. *286*, 27875–27881. https://doi.org/10.1074/jbc.C110.216580.
8. Lu, Y.Y., Hao, B.X., Graeff, R., Wong, C.W.M., Wu, W.T., and Yue, J. (2017). Two pore channel 2 (TPC2) inhibits autophagosomal-lysosomal fusion by alkalinizing lysosomal pH. J. Biol. Chem. *292*, 12088. https://doi.org/10.1074/jbc.A113.484253.
9. Alharbi, A.F., and Parrington, J. (2021). TPC2 targeting evolution: Leveraging therapeutic opportunities for cancer. Cell Chem. Biol. *28*, 1103–1105. https://doi.org/10.1016/j.chembiol.2021.07.020.
10. Li, Y., Schön, C., Chen, C.C., Yang, Z., Liegl, R., Murenu, E., Schworm, B., Klugbauer, N., Grimm, C., Wahl-Schott, C., et al. (2021). TPC2 promotes choroidal angiogenesis and inflammation in a mouse model of neovascular age-related macular degeneration. Life Sci. Alliance *4*, e202101047. https://doi.org/10.26508/lsa.202101047.
11. Heister, P.M., and Poston, R.N. (2020). Pharmacological hypothesis: TPC2 antagonist tetrandrine as a potential therapeutic agent for COVID-19. Pharmacol. Res. Perspect. *8*, e00653. https://doi.org/10.1002/prp2.653.
12. Müller, M., Gerndt, S., Chao, Y.K., Zisis, T., Nguyen, O.N.P., Gerwien, A., Urban, N., Müller, C., Gegenfurtner, F.A., Geisslinger, F., et al. (2021). Gene editing and synthetically accessible inhibitors reveal role for TPC2 in HCC cell proliferation and tumor growth. Cell Chem. Biol. *28*, 1119–1131.e1127. https://doi.org/10.1016/j.chembiol.2021.01.023.
13. Pafumi, I., Festa, M., Papacci, F., Lagostena, L., Giunta, C., Gutla, V., Cornara, L., Favia, A., Palombi, F., Gambale, F., et al. (2017). Naringenin Impairs Two-Pore Channel 2 Activity And Inhibits VEGF-Induced Angiogenesis. Sci. Rep. *7*, 5121. https://doi.org/10.1038/s41598-017-04974-1.
14. D'Amore, A., Hanbashi, A.A., Di Agostino, S., Palombi, F., Sacconi, A., Voruganti, A., Taggi, M., Canipari, R., Blandino, G., Parrington, J., and Filippini, A. (2020). Loss of Two-Pore Channel 2 (TPC2) Expression Increases the Metastatic Traits of Melanoma Cells by a Mechanism Involving the Hippo Signalling Pathway and Store-Operated Calcium Entry. Cancers *12*, 2391. https://doi.org/10.3390/cancers12092391.
15. Alharbi, A.F., and Parrington, J. (2019). Endolysosomal Ca(2+) Signaling in Cancer: The Role of TPC2, From Tumorigenesis to Metastasis. Front. Cell Dev. Biol. *7*, 302. https://doi.org/10.3389/fcell.2019.00302.
16. Grimm, C., Holdt, L.M., Chen, C.C., Hassan, S., Müller, C., Jörs, S., Cuny, H., Kissing, S., Schröder, B., Butz, E., et al. (2014). High susceptibility to fatty liver disease in two-pore channel 2-deficient mice. Nat. Commun. *5*, 4699. https://doi.org/10.1038/ncomms5699.
17. Böck, J., Krogsaeter, E., Passon, M., Chao, Y.K., Sharma, S., Grallert, H., Peters, A., and Grimm, C. (2021). Human genome diversity data reveal that L564P is the predominant TPC2 variant and a prerequisite for the blond hair associated M484L gain-of-function effect. PLoS Genet. *17*, e1009236. https://doi.org/10.1371/journal.pgen.1009236.
18. Chao, Y.K., Schludi, V., Chen, C.C., Butz, E., Nguyen, O.N.P., Müller, M., Krüger, J., Kammerbauer, C., Ben-Johny, M., Vollmar, A.M., et al. (2017). TPC2 polymorphisms associated with a hair pigmentation phenotype in humans result in gain of channel function by independent mechanisms. Proc. Natl. Acad. Sci. USA *114*, E8595–E8602. https://doi.org/10.1073/pnas.1705739114.
19. Zhao, Z., Qin, P., and Huang, Y.W. (2021). Lysosomal ion channels involved in cellular entry and uncoating of enveloped viruses: Implications for therapeutic strategies against SARS-CoV-2. Cell Calcium *94*, 102360. https://doi.org/10.1016/j.ceca.2021.102360.
20. Chao, Y.K., Chang, S.Y., and Grimm, C. (2023). Endo-Lysosomal Cation Channels and Infectious Diseases. Rev. Physiol. Biochem. Pharmacol. *185*, 259–276. https://doi.org/10.1007/112_2020_31.
21. Grimm, C., and Tang, R. (2020). Could an endo-lysosomal ion channel be the Achilles heel of SARS-CoV2? Cell Calcium *88*, 102212. https://doi.org/10.1016/j.ceca.2020.102212.
22. Penny, C.J., Vassileva, K., Jha, A., Yuan, Y., Chee, X., Yates, E., Mazzon, M., Kilpatrick, B.S., Muallem, S., Marsh, M., et al. (2019). Mining of Ebola virus entry inhibitors identifies approved drugs as two-pore channel pore blockers. Biochim. Biophys. Acta. Mol. Cell Res. *1866*, 1151–1161. https://doi.org/10.1016/j.bbamcr.2018.10.022.
23. Ayele, A.G., Enyew, E.F., and Kifle, Z.D. (2021). Roles of existing drug and drug targets for COVID-19 management. Metabol. Open *11*, 100103. https://doi.org/10.1016/j.metop.2021.100103.
24. Gerndt, S., Krogsaeter, E., Patel, S., Bracher, F., and Grimm, C. (2020). Discovery of lipophilic two-pore channel agonists. FEBS J. *287*, 5284–5293. https://doi.org/10.1111/febs.15432.
25. Naylor, E., Arredouani, A., Vasudevan, S.R., Lewis, A.M., Parkesh, R., Mizote, A., Rosen, D., Thomas, J.M., Izumi, M., Ganesan, A., et al. (2009). Identification of a chemical probe for NAADP by virtual screening. Nat. Chem. Biol. *5*, 220–226. https://doi.org/10.1038/nchembio.150.
26. Kirsch, S.A., Kugemann, A., Carpaneto, A., Böckmann, R.A., and Dietrich, P. (2018). Phosphatidylinositol-3,5-bisphosphate lipid-binding-induced activation of the human two-pore channel 2. Cell. Mol. Life Sci. *75*, 3803–3815. https://doi.org/10.1007/s00018-018-2829-5.
27. Zhang, J., Guan, X., Shah, K., and Yan, J. (2021). Lsm12 is an NAADP receptor and a two-pore channel regulatory protein required for calcium mobilization from acidic organelles. Nat. Commun. *12*, 4739. https://doi.org/10.1038/s41467-021-24735-z.
28. Roggenkamp, H.G., Khansahib, I., Hernandez, C.L., Zhang, Y., Lodygin, D., Krüger, A., Gu, F., Möckl, F., Löhndorf, A., Wolters, V., et al. (2021). HN1L/JPT2: A signaling protein that connects NAADP generation to Ca(2+) microdomain formation. Sci. Signal. *14*. https://doi.org/10.1126/scisignal.abd5647.
29. Gunaratne, G.S., Brailoiu, E., He, S., Unterwald, E.M., Patel, S., Slama, J.T., Walseth, T.F., and Marchant, J.S. (2021). Essential requirement for JPT2 in NAADP-evoked Ca(2+) signaling. Sci. Signal. *14*, eabd5605. https://doi.org/10.1126/scisignal.abd5605.
30. Patel, S., Yuan, Y., Gunaratne, G.S., Rahman, T., and Marchant, J.S. (2022). Activation of endo-lysosomal two-pore channels by NAADP and PI(3,5)P(2). Five things to know. Cell Calcium *103*, 102543. https://doi.org/10.1016/j.ceca.2022.102543.

CellPress
OPEN ACCESS

31. Krogsaeter, E., Tang, R., and Grimm, C. (2021). JPT2: The missing link between intracellular Ca(2+) release channels and NAADP? Cell Calcium *97*, 102405. https://doi.org/10.1016/j.ceca.2021.102405.

32. Gerndt, S., Chen, C.C., Chao, Y.K., Yuan, Y., Burgstaller, S., Scotto Rosato, A., Krogsaeter, E., Urban, N., Jacob, K., Nguyen, O.N.P., et al. (2020). Agonist-mediated switching of ion selectivity in TPC2 differentially promotes lysosomal function. Elife *9*, e54712. https://doi.org/10.7554/eLife.54712.

33. Sakurai, Y., Kolokoltsov, A.A., Chen, C.-C., Tidwell, M.W., Bauta, W.E., Klugbauer, N., Grimm, C., Wahl-Schott, C., Biel, M., and Davey, R.A. (2015). Two-pore channels control Ebola virus host cell entry and are drug targets for disease treatment. Science *347*, 995–998. https://doi.org/10.1126/science.1258758.

34. Netcharoensirisuk, P., Abrahamian, C., Tang, R., Chen, C.C., Rosato, A.S., Beyers, W., Chao, Y.K., Filippini, A., Di Pietro, S., Bartel, K., et al. (2021). Flavonoids increase melanin production and reduce proliferation, migration and invasion of melanoma cells by blocking endolysosomal/melanosomal TPC2. Sci. Rep. *11*, 8515. https://doi.org/10.1038/s41598-021-88196-6.

35. She, J., Zeng, W., Guo, J., Chen, Q., Bai, X.C., and Jiang, Y. (2019). Structural mechanisms of phospholipid activation of the human TPC2 channel. Elife *8*, e45222. https://doi.org/10.7554/eLife.45222.

36. Kintzer, A.F., Green, E.M., Dominik, P.K., Bridges, M., Armache, J.-P., Deneka, D., Kim, S.S., Hubbell, W., Kossiakoff, A.A., Cheng, Y., and Stroud, R.M. (2018). Structural basis for activation of voltage sensor domains in an ion channel TPC1. Proc. Natl. Acad. Sci. USA *115*, E9095–E9104. https://doi.org/10.1073/pnas.1805651115.

37. Zhang, X., Chen, W., Li, P., Calvo, R., Southall, N., Hu, X., Bryant-Genevier, M., Feng, X., Geng, Q., Gao, C., et al. (2019). Agonist-specific voltage-dependent gating of lysosomal two-pore Na(+) channels. Elife *8*, e51423. https://doi.org/10.7554/eLife.51423.

38. Ahuja, S., Mukund, S., Deng, L., Khakh, K., Chang, E., Ho, H., Shriver, S., Young, C., Lin, S., Johnson, J.P., et al. (2015). Structural basis of Nav1.7 inhibition by an isoform-selective small-molecule antagonist. Science *350*, aac5464. https://doi.org/10.1126/science.aac5464.

39. Zheng, Y., Liu, H., Chen, Y., Dong, S., Wang, F., Wang, S., Li, G.-L., Shu, Y., and Xu, F. (2022). Structural insights into the lipid and ligand regulation of a human neuronal KCNQ channel. Neuron *110*, 237–247.e4. https://doi.org/10.1016/j.neuron.2021.10.029.

40. Botte, M., Huber, S., Bucher, D., Klint, J.K., Rodríguez, D., Tagmose, L., Chami, M., Cheng, R., Hennig, M., and Abdul Rahman, W. (2022). Apo and ligand-bound high resolution Cryo-EM structures of the human Kv3.1 channel reveal a novel binding site for positive modulators. PNAS Nexus *1*, pgac083. https://doi.org/10.1093/pnasnexus/pgac083.

41. Dickinson, M.S., Lu, J., Gupta, M., Marten, I., Hedrich, R., and Stroud, R.M. (2022). Molecular basis of multistep voltage activation in plant two-pore channel 1. Proc. Natl. Acad. Sci. USA *119*, e2110936119. https://doi.org/10.1073/pnas.2110936119.

42. She, J., Guo, J., Chen, Q., Zeng, W., Jiang, Y., and Bai, X.C. (2018). Structural insights into the voltage and phospholipid activation of the mammalian TPC1 channel. Nature *556*, 130–134. https://doi.org/10.1038/nature26139.

43. Tuluc, P., Yarov-Yarovoy, V., Benedetti, B., and Flucher, B.E. (2016). Molecular Interactions in the Voltage Sensor Controlling Gating Properties of Ca_V Calcium Channels. Structure *24*, 261–271. https://doi.org/10.1016/j.str.2015.11.011.

44. Labro, A.J., Priest, M.F., Lacroix, J.J., Snyders, D.J., and Bezanilla, F. (2015). Kv3.1 uses a timely resurgent K(+) current to secure action potential repolarization. Nat. Commun. *6*, 10173. https://doi.org/10.1038/ncomms10173.

45. Priest, M.F., Lacroix, J.J., Villalba-Galea, C.A., and Bezanilla, F. (2013). S3-S4 Linker Length Modulates the Relaxed State of a Voltage-Gated Potassium Channel. Biophys. J. *105*, 2312–2322. https://doi.org/10.1016/j.bpj.2013.09.053.

46. Shimomura, T., Hirazawa, K., and Kubo, Y. (2023). Conformational rearrangements in the second voltage sensor domain switch PIP(2)- and voltage-gating modes in two-pore channels. Proc. Natl. Acad. Sci. USA *120*, e2209569120. https://doi.org/10.1073/pnas.2209569120.

47. Ye, F., Xu, L., Li, X., Zeng, W., Gan, N., Zhao, C., Yang, W., Jiang, Y., and Guo, J. (2021). Voltage-gating and cytosolic Ca2+ activation mechanisms of Arabidopsis two-pore channel AtTPC1. Proc. Natl. Acad. Sci. USA *118*, e2113946118. https://doi.org/10.1073/pnas.2113946118.

48. Alharbi, A.F., and Parrington, J. (2021). The role of genetic polymorphisms in endolysosomal ion channels TPC2 and P2RX4 in cancer pathogenesis, prognosis, and diagnosis: a genetic association in the UK Biobank. NPJ Genom. Med. *6*, 58. https://doi.org/10.1038/s41525-021-00221-9.

49. Jiang, D., Tonggu, L., Gamal El-Din, T.M., Banh, R., Pomès, R., Zheng, N., and Catterall, W.A. (2021). Structural basis for voltage-sensor trapping of the cardiac sodium channel by a deathstalker scorpion toxin. Nat. Commun. *12*, 128. https://doi.org/10.1038/s41467-020-20078-3.

50. Xu, H., Li, T., Rohou, A., Arthur, C.P., Tzakoniati, F., Wong, E., Estevez, A., Kugel, C., Franke, Y., Chen, J., et al. (2019). Structural Basis of Nav1.7 Inhibition by a Gating-Modifier Spider Toxin. Cell *176*, 702–715.e14. https://doi.org/10.1016/j.cell.2018.12.018.

51. Kintzer, A.F., and Stroud, R.M. (2018). On the structure and mechanism of two-pore channels. FEBS J. *285*, 233–243. https://doi.org/10.1111/febs.14154.

52. Punjani, A., Rubinstein, J.L., Fleet, D.J., and Brubaker, M.A. (2017). cryoSPARC: algorithms for rapid unsupervised cryo-EM structure determination. Nat. Methods *14*, 290–296. https://doi.org/10.1038/nmeth.4169.

53. Punjani, A., and Fleet, D.J. (2021). 3D variability analysis: Resolving continuous flexibility and discrete heterogeneity from single particle cryo-EM. J. Struct. Biol. *213*, 107702. https://doi.org/10.1016/j.jsb.2021.107702.

54. Rohou, A., and Grigorieff, N. (2015). CTFFIND4: Fast and accurate defocus estimation from electron micrographs. J. Struct. Biol. *192*, 216–221. https://doi.org/10.1016/j.jsb.2015.08.008.

55. Liebschner, D., Afonine, P.V., Baker, M.L., Bunkóczi, G., Chen, V.B., Croll, T.I., Hintze, B., Hung, L.-W., Jain, S., McCoy, A.J., et al. (2019). Macromolecular structure determination using X-rays, neutrons and electrons: recent developments in Phenix. Acta Crystallogr. D Struct. Biol. *75*, 861–877. https://doi.org/10.1107/S2059798319011471.

56. Long, F., Nicholls, R.A., Emsley, P., Graǽulis, S., Merkys, A., Vaitkus, A., and Murshudov, G.N. (2017). AceDRG: a stereochemical description generator for ligands. Acta Crystallogr. D Struct. Biol. *73*, 112–122. https://doi.org/10.1107/S2059798317000067.

57. Mahajan, P., Ellis, K., Mukhopadhyay, S., Fernandez-Cid, A., Chi, G., Man, H., Dürr, K.L., and Burgess-Brown, N.A. (2021). Expression Screening of Human Integral Membrane Proteins Using BacMam. In Structural Genomics: General Applications, Y.W. Chen and C.-P.B. Yiu, eds. (Springer US), pp. 95–115. https://doi.org/10.1007/978-1-0716-0892-0_6.

58. Emsley, P., Lohkamp, B., Scott, W.G., and Cowtan, K. (2010). Features and development of Coot. Acta Crystallogr. D *66*, 486–501. https://doi.org/10.1107/S0907444910007493.

59. Williams, C.J., Headd, J.J., Moriarty, N.W., Prisant, M.G., Videau, L.L., Deis, L.N., Verma, V., Keedy, D.A., Hintze, B.J., Chen, V.B., et al. (2018). MolProbity: More and better reference data for improved all-atom structure validation. Protein Sci. *27*, 293–315. https://doi.org/10.1002/pro.3330.

STAR★METHODS

KEY RESOURCES TABLE

REAGENT or RESOURCE	SOURCE	IDENTIFIER
Chemicals, peptides, and recombinant proteins		
β-dodecyl maltopyranoside	Generon	Cat#D310LA
Cholesteryl hemisuccinate	Merck	Cat#C6512
Glyco-diosgenin	Generon	Cat#GDN101
Strep-Tactin Superflow	IBA Lifesciences	Cat#2-1206-025
D-desthiobiotin	Sigma-Aldrich	Cat#D1411-1G
Prometheus NT.48 Series nanoDSF Grade Standard Capillaries	NanoTemper	Cat#PR-C002
Quantifoil Au R1.2/1.3 300-mesh grid	Quantifoil	Cat#N1-C14nAu30-01
FastDigest DpnI	Thermo Fisher Scientific	Cat#FD1703
Fura-2 a.m.	Abcr GmbH	Cat#AB348887
Turbofect	Thermo Fisher Scientific	Cat#R0532
TPC2-A1-N	This paper, Müller et al.[32]	N/A
TPC2-A1-P	This paper, Müller et al.[32]	N/A
SG-094	This paper, Müller et al.[12]	N/A
(R)-SG-094	This paper, Müller et al.[12]	N/A
(S)-SG-094	This paper, Müller et al.[12]	N/A
Critical commercial assays		
KAPA HiFi HotStart ReadyMix Mutagenesis kit	Roche	Cat#KK2601
Compact Prep Plasmid Mini Kit	Invitrogen	Cat#K210010
Deposited data		
Cryo-EM map for *Hs*TPC2 with *(S)*-SG-094	This paper	EMDB: EMD-17197
Structure model for *Hs*TPC2 with *(S)*-SG-094	This paper	PDB: 8OUO
Cryo-EM map for *Hs*TPC2 with *(R)*-SG-094	This paper	EMDB: EMD-19108
Experimental models: Cell lines		
Sf9 cells	Thermo Fisher Scientific	Cat#11496015
Expi293F^{TM} GnTI^{-} cells	Thermo Fisher Scientific	Cat#A39240
HEK293 cells	ATCC	Cat#CRL-1573; RRID: CVCL_0045
Oligonucleotides		
TPC2^{N439A} forward primer 5′ GACTACCTGGGGGCGCTCATCG CCCTGGC 3′	This paper	N/A
TPC2^{N439A} reverse primer 5′ GCCAGGGCGATGAGCGCCCCCA GGTAGTC 3′	This paper	N/A
TPC2^{F555A} forward primer 5′ CATCGTGTTCCGCGCGCTGCGT ATCATCC 3′	This paper	N/A
TPC2^{F555A} reverse primer 5′ GGATGATACGCAGCGCGCGGA ACACGATG 3′	This paper	N/A
Recombinant DNA		
pHTBV1.1-TPC2 plasmid	This paper	N/A
pHTBV1.1-TPC2^{Y436A} plasmid	Twist Bioscience	N/A
pHTBV1.1-TPC2^{F555E} plasmid	Twist Bioscience	N/A

(*Continued on next page*)

Continued		
REAGENT or RESOURCE	SOURCE	IDENTIFIER
TPC2^{Y436A} gene	Genscript	N/A
Software and algorithms		
EPU	Thermo Fisher Scientific	N/A
CryoSPARC v2.11	Structura Biotechnology, Punjani et al.[52,53]	N/A
CTFFIND4.11.0	Rohou et al.[54]	N/A
Phenix1.20.1	Liebschner et al.[55]	N/A
Acedrg	Long et al.[56]	N/A
Prometheus ThermControl	NanoTemper	N/A
LeDock	LePhar	http://www.lephar.com/software.htm
PatchMaster	HEKA Elektronik	N/A
Other		
Fetal bovine serum	Thermo Fisher Scientific	Cat#A5670701
Penicillin-Streptomycin	Thermo Fisher Scientific	Cat#15070063
Sf-900™ II medium	Thermo Fisher Scientific	Cat#10902096
Dulbecco's Modified Eagle Medium (DMEM)	Thermo Fisher Scientific	Cat#11885084
Freestyle 293™ Expression medium	Thermo Fisher Scientific	Cat#12338018

RESOURCE AVAILABILITY

Lead contact

Further information and requests for resources and reagents should be directed to and will be fulfilled by the lead contact, Gamma Chi (gamma.chi@cmd.ox.ac.uk).

Materials availability

All unique reagents generated in this study are available from the lead contact with a completed Materials Transfer Agreement.

Data and code availability

- Cryo-EM maps and models have been deposited to Protein DataBank, and are publicly accessible. *Hs*TPC2/*(S)*-SG-094 structure map and model have the following accession code: EMD-17197 (EMDB), 8OUO (PDB). *Hs*TPC2/*(R)*-SG-094 structure map has the following accession code: EMD-19108 (EMDB).
- This paper does not report original code.
- Any additional information required to reanalyse the data reported is available from the lead contact upon request.

EXPERIMENTAL MODEL AND STUDY PARTICIPANT DETAILS

HEK293 cells were obtained from the American Type Culture Collection (ATCC, cat: # CRL-1573), and maintained in Dulbecco's Modified Eagle Medium (DMEM, Thermo Fisher Scientific, cat: # 12338018) supplemented with 100 U/mL penicillin, 100 g/mL streptomycin (Thermo Fisher Scientific, cat: # 15070063) and 10% FBS (Thermo Fisher Scientific, cat: # 5670701) at 37°C Expi293F GnTI^{-} cells were obtained from Thermo Fisher Scientific (cat: # A39240), and maintained in Freestyle 293TM Expression Medium (Thermo Fisher Scientific, cat: # 12338018) at 37°C Sf9 cells were obtained from Thermo Fisher Scientific (cat: # 11496015), and maintained in Sf-900 II media. *Escherichia coli* DH10Bac cells were obtained from Thermo Fisher Scientific (cat: # 10361012), and grown in LB media at 37°C.

METHOD DETAILS

Molecular biology, virus production and protein expression

Full-length *Hs*TPC2 with L11A/L12A mutations for plasma membrane targeting and its point mutation variants (Y436A, F555E) cloned into a pHTBV N-terminally tagged twin-Strep, 10-His vector with GFP were synthesized (Twist Bioscience).

Baculoviruses for these constructs were generated following the standard protocol outlined in.[57] *Escherichia coli* DH10Bac cells were transformed with plasmids containing the *Hs*TPC2 genes. Baculoviral DNA extracted from the cells were used to transfect Sf9 cells grown in Sf-900 II media supplemented with 2% fetal bovine serum (Thermo Fisher Scientific) and incubated on an orbital shaker

for 70 h at 27°C. Produced baculovirus particles were harvested by centrifugation at 900*g* for 10 min and collecting the supernatants, and they were further amplified with Sf9 cells.

Each liter of Expi293F GnTI$^-$ cell culture in Freestyle 293 Expression Medium (Thermo Fisher Scientific) were infected with 30 mL of P3 baculovirus-containing supernatant in the presence of 5 mM sodium butyrate. Cells were grown in an orbital shaker for 70 h at 30°C and 8% CO_2 before being harvested by centrifugation at 900*g* for 10 min, washed with phosphate-buffered saline, then centrifuged again. The cell washed cell pellets were flash-frozen with liquid nitrogen (LN_2), then stored at −80°C until needed.

SG-094 compound synthesis and preparation

Racemic SG-094 was synthesized and its *(S)*- and *(R)*- enantiomers were purified as previously described.[12] The compounds were dissolved in DMSO to a final concentration of 50 mM. The compound stocks were stored at −20°C for use within two months.

Protein purification

For the purification of *Hs*TPC2 for thermal shift assays, the following protocol was used. Whole cell pellets expressing *Hs*TPC2 constructs were resuspended to a total volume of 50 mL per 15 g of cell pellet with buffer A (20 mM HEPES pH 7.5, 150 mM NaCl) supplemented with 0.7% β-dodecyl maltopyranoside (β-DDM; Generon) and 0.07% cholesteryl hemisuccinate (CHS; Merck). The cells were solubilized at 4°C for 1 h with gentle rotation. Cell debris was pelleted by centrifugation at 45,000*g* for 1 h. The clarified lysate was added to 0.5 mL bed volume of Strep-Tactin Superflow (IBA) per 100 mL of lysate, and allowed to bind at 4°C for 1 h. The resin was collected on a gravity-flow column and washed with buffer B (buffer A with 0.02% β-DDM and 0.002% CHS), then with buffer B supplemented with 2 mM ATP and 5 mM $MgCl_2$. Protein was eluted with 10 CV of buffer B containing 5 mM D-desthiobiotin followed by tag cleavage by Tobacco Etch Virus protease overnight and reverse purification. The samples were subjected to size exclusion chromatography with a Superose 6 Increase 10/300 column (GE Healthcare) pre-equilibrated with buffer C (buffer A with 0.01% glyco-diosgenin, GDN, Generon). Peak fractions were pooled and concentrated to 1 μM.

For the purification of *(S)*-SG-094-bound *Hs*TPC2 for cryo-electron microscopy, the method described above was used, with buffer A consisting of 20 mM HEPES pH 7.5, 150 mM NaCl and 5 μM *(S)*-SG-094 instead. After pooling fractions from size exclusion chromatography, *(S)*-SG-094 in DMSO was added to the sample to a final concentration of 200 μM, followed by overnight incubation on a rotating wheel at 4°C. The sample was concentrated to 50 μM and used immediately for cryo-EM sample preparation.

nanoDSF thermal shift assay

20x stock solutions of the tested compounds were prepared by diluting DMSO-solubilised initial stocks to 1 mM in buffer C. Purified *Hs*TPC2 constructs and the compounds were mixed and incubated on ice for 4 h. Prometheus NT.48 Series nanoDSF Grade Standard Capillaries (NanoTemper) were loaded with 10 μL of samples, and melting curves were determined in triplicates using Prometheus NT.48 (NanoTemper) by monitoring intrinsic tryptophan fluorescence signals over a temperature range from 20°C to 95°C with 1°C/min ramp. The melting temperature of each condition was determined by averaging the melting temperatures of the triplicate measurements.

Cryo-EM sample preparation, data collection and processing

Samples were frozen on Quantifoil Au R1.2/1.3 300-mesh grids glow-discharged for 30 s, with plunge freezing performed on Vitrobot Mark IV (Thermo Fisher Scientific) set to 100% humidity and 4°C.

The cryo-EM dataset was collected on a Titan Krios (Thermo Fisher Scientific) operating at 300 keV at eBIC (Didcot, UK). 9,924 super-resolution dose-fractionated micrographs (0.4145 Å $pixel^{-1}$) were collected on a K3 detector at 105,000× nominal magnification by aberration-free image shift (AFIS) collection mode, with a total dose of 38 $e^-.A^{-2}$ over 31 frames.

Micrographs were imported to Cryosparc v2.11 and motion-corrected with its Patch Motion Correction function.[52] After defocus estimation with CTFFIND 4.11.0 [54], 2,508,787 particles were picked with blob-picking function and extracted to 0.829 Å $pixel^{-1}$ with 300 pixel box size. Iterative 2D classifications led to 751,050 polished hsTPC2 particles, of which 20,000 were used to build an ab initio reconstruction. The full set of particles were then subjected to heterogeneous refinement, where 432,072 further polished particles were isolated. These were then used for a 3D refinement with C1 symmetry, which resulted in an electrostatic potential (ESP) map of 2.86 Å nominal resolution. The refined particles were subjected to 3D variability analysis with four modes using a mask generated from unsharpened refined map. Cluster function was used to isolate particles for a subclass with 109,417 particles clearly showing alternate state of voltage-sensing domain (VSD). These particles were used for a new 3D refinement, which resulted in an ESP map of 2.98 Å nominal resolution.

Model building and refinement

*Hs*TPC2 structure model in apo state (PDB ID: 6NQ1) was fitted to the ESP map of SG-094-bound *Hs*TPC2 and used as template for manual refinement in Coot.[58] The model was refined with Phenix real space refine[55] and geometry of the models were verified with MolProbity function in Phenix.[59] Model coordinates and restraints for SG-094 were generated using Acedrg.[56] SG-094 model was fitted into ESP feature consistent with the compound.

Compound docking simulation

Compound docking simulation using LeDock (LePhar) was performed to validate the binding mode of SG-094. PDB coordinates of hsTPC2 in *(S)*-SG-094-bound state and MDL molfile of *(S)*-SG-094 molecule were loaded as inputs, and a cubic search area of 25 Å × 25 Å x 25 Å was defined around the binding pocket. PDB coordinates of ligand outputs were manually assessed and compared with experimentally determined binding pose of SG-094.

Site-directed mutagenesis, transformation and isolation of plasmid DNA

The following primer sequences for site-directed mutagenesis were used to generate $TPC2^{N439A}$: GACTACCTGGGGGCG CTCATCGCCCTGGC (forward), GCCAGGGCGATGAGCGCCCCCAGGTAGTC (reverse). The primer sequences for $TPC2^{F555A}$ mutant were CATCGTGTTCCGCGCGCTGCGTATCATCC (forward), GGATGATACGCAGCGCGCGGAACACGATG (reverse). Procedure for KAPA HiFi HotStart ReadyMix Mutagenesis kit (Roche): 50 ng of plasmid DNA and 295 nM for each forward and reverse primers were used in a 50 μL reaction. PCR was done with a Mastercycler nexus gradient (Eppendorf). PCR conditions applied: initial denaturation at 95°C for 5 min, then denaturation at 98°C for 20 s, followed by annealing step at 56°C for 1 min and an elongation step at 72°C for 10 min with an additional final elongation step at 72°C for 9 min. After PCR amplification, the mix was digested for 1 h at 37°C with FastDigest DpnI. Subsequently, 10-beta competent *E. coli* cells were used for transformation. For transformation competent *E. coli* cells were thawed on ice for 10 min. Then, 5 μL of the ligation was added to the cells, which corresponds to 10% of the total volume of the cells. Then, the mixture was placed back on ice for 30 min and the cells were subjected to heat shock at 42°C for 45 s. Afterward, the tube was placed on ice for 5 min and 500 μL of preheated LB(+) medium was added. The sample grew at 37°C for 60 min at 180 rpm in an incubator. Finally, samples were spread onto selection plates and incubated overnight at 37°C. After colony growth, single colonies were picked from each plate and cultured for 16 h at 37 °C at 200 rpm in 4 mL LB(+) medium with 100 μg/mL ampicillin. The plasmid DNA was isolated using a Compact Prep Plasmid Mini Kit (Invitrogen). All clones were sequence verified and the point mutations confirmed. The $TPC2^{Y436A}$ mutation was obtained from GenScript, who used the cloneEZ method to create the single amino acid substitution.

Cell culture, transient transfection, Ca^{2+} imaging experiments and whole cell patch-clamp electrophysiology

HEK293 cells were cultured in Dulbecco's Modified Eagle Medium (DMEM) supplemented with 100 U/mL penicillin, 100 g/mL streptomycin and 10% FBS at 37°C in a humidified chamber at 95% air and 5% CO_2. HEK293 cells were seeded on 25 mm^2 glass coverslips in Standard TC plates (6-well plate) at a density of $2 \cdot 10^5$ per well. The cells grew for 48 h and were subsequently transfected. The transfection was performed using 1.5 μg of total DNA, 3 μL of Turbofect (Thermo Fisher Scientific), and 200 μL of serum-free DMEM per one well. The cells were then incubated at 37°C for 24 h. For Ca^{2+} imaging experiments, the transfected HEK293 cells were washed in Ca^{2+} buffer containing 138 mM NaCl, 6 mM KCl, 2 mM $MgCl_2$, 2 mM $CaCl_2$, 10 mM HEPES, and 5.5 mM D-glucose (adjusted to pH 7.4 with NaOH). Thereafter, each well of the 6-well plate was loaded with Fura-2 a.m. (4.0 mM) and 0.005% (v/v) Pluronic acid (stock solution at 10%), both diluted in Ca^{2+} buffer with a volume of 1 mL per well. The 6-well plate with cells was incubated at 37°C for 45 min. After incubation, wells were carefully washed twice with 1 mL of Ca^{2+} buffer per well. Then, each coverslip with the cells was placed into the imaging chamber and 450 μL of Ca^{2+} buffer was added to the chamber slowly. The osmolarity of the Ca^{2+} buffer was 300 mOsmol/L. Ca^{2+} imaging was performed using an inverted Leica DMi8 live cell microscope. Fura-2 was excited at two wavelengths: 340 nm/387 nm. Emitted fluorescence was captured using a 515 nm long-pass filter. Compounds were diluted in DMSO and stored as 10 mM stock solutions. Working solutions were prepared with Ca^{2+} buffer directly before usage. Manual patch-clamp recordings on transiently transfected HEK cells (as described above) were conducted in whole cell configuration. Bath solution was identical to the Ca^{2+} buffer described above and pipette solution contained 140 mM K-MSA, 5 mM KOH, 4 mM NaCl, 0.39 mM $CaCl_2$, 1 mM EGTA and 20 mM HEPES (pH was adjusted with KOH to 7.2). For small molecule application, cytoplasmic solution was completely exchanged by cytoplasmic solution containing agonist (TPC2-A1-N), freshly diluted before the experiment. SG-094 (10 μM) was added subsequently to block activated hTPC2 channels. Recording glass pipettes were pulled and polished to resistances in the range of 3–4 MΩ. Electrophysiological recordings were performed with an EPC10 patch-clamp amplifier (HEKA, Lambrecht, Germany), operated by PatchMaster software (HEKA Elektronik). Fast and slow capacitive transients were canceled by the compensation circuit of the EPC-10 amplifier. In all experiments, 500 ms voltage ramps from +100 to −100 mV were applied every 5 s, holding potential was kept at +60 mV. Digitized and filtered (40 kHz and low-pass filter frequency of 2.9 kHz) current amplitudes at −100 mV were extracted from individual ramp current recordings.

QUANTIFICATION AND STATISTICAL ANALYSIS

All plots for cell-based assays (Figures 4, 5, and S6) were made with GraphPad Prism 9.0.2, and statistical analysis details can be found in the respective figure legends. Results for Ca^{2+} assays (Figure 4) were analyzed with unpaired t test and expressed as mean ± SEM in figures. Results for electrophysiological recordings (Figures 5 and S6) were analyzed with one-way ANOVA, Tukey's post hoc test. Each biological data point represents an average of technical replicates of 5–10 cells. Number of biological replicates (*n* values) are detailed in the legends for each figure.

Results for thermal shift assays (Figures S2 and S5) were analyzed with Prometheus NT.48 to calculate melting temperatures, and mean values for triplicate experiments were calculated with Excel 2013.Cryo-EM data collection and refinement statistics are reported in Table S1.

Structure, Volume *32*

Supplemental Information

Structural basis for inhibition of the lysosomal two-pore channel TPC2 by a small molecule antagonist

Gamma Chi, Dawid Jaślan, Veronika Kudrina, Julia Böck, Huanyu Li, Ashley C.W. Pike, Susanne Rautenberg, Einar Krogsaeter, Tina Bohstedt, Dong Wang, Gavin McKinley, Alejandra Fernandez-Cid, Shubhashish M.M. Mukhopadhyay, Nicola A. Burgess-Brown, Marco Keller, Franz Bracher, Christian Grimm, and Katharina L. Dürr

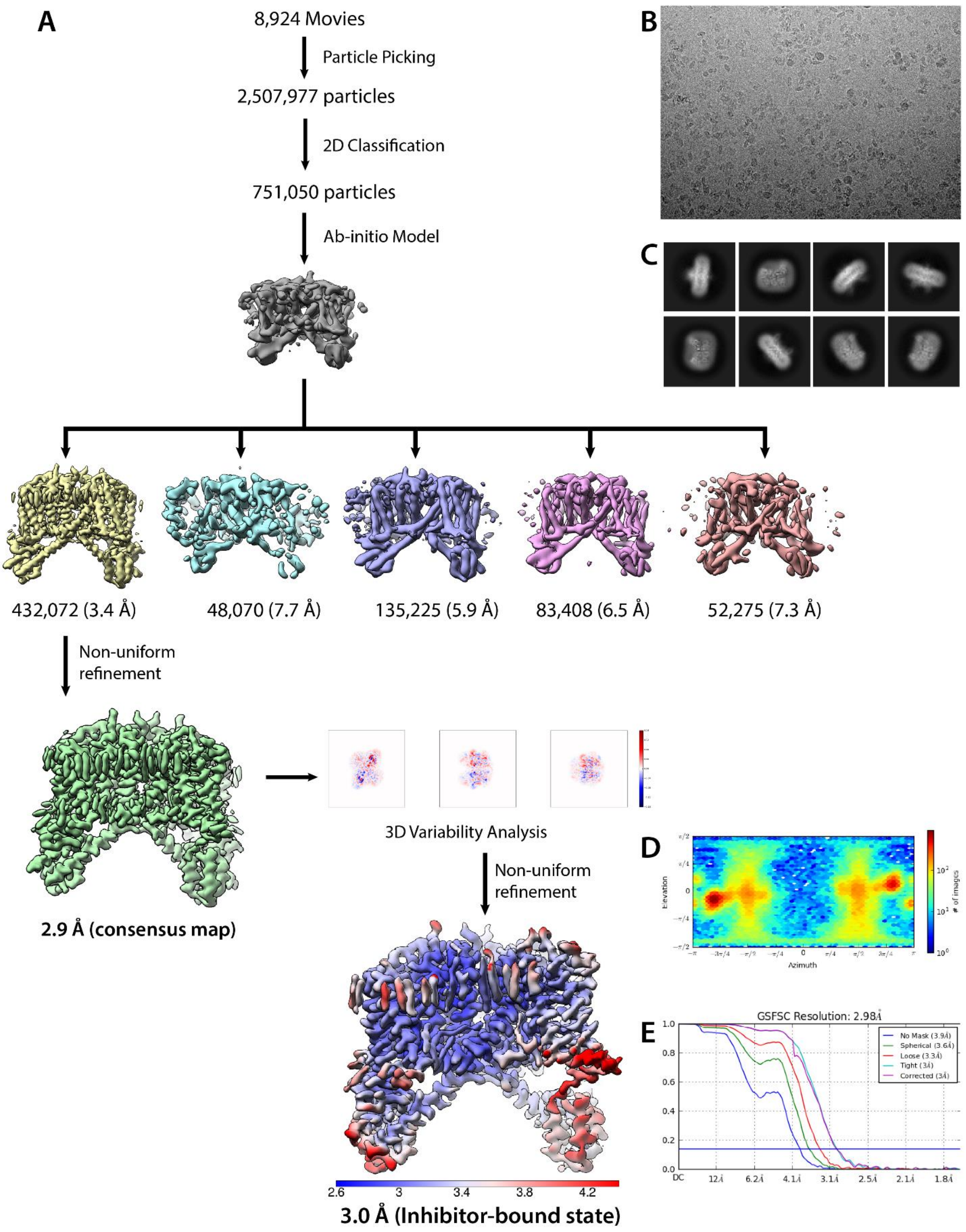

Supplementary Figure S1. Flowchart of structure determination with cryo-electron microscopy for *Hs*TPC2 in complex with small molecule antagonist *(S)*-SG-094, related to Figure 1 and STAR Methods (Cryo-EM Sample Preparation, Data Collection and Processing).

A) Data processing workflow. **B)** Sample raw micrograph of the collected dataset. **C)** Representative 2D classes of the collected dataset. **D)** Orientation distribution of the final reconstruction. **E)** Fourier Shell Correlation (FSC) curve of the final reconstruction

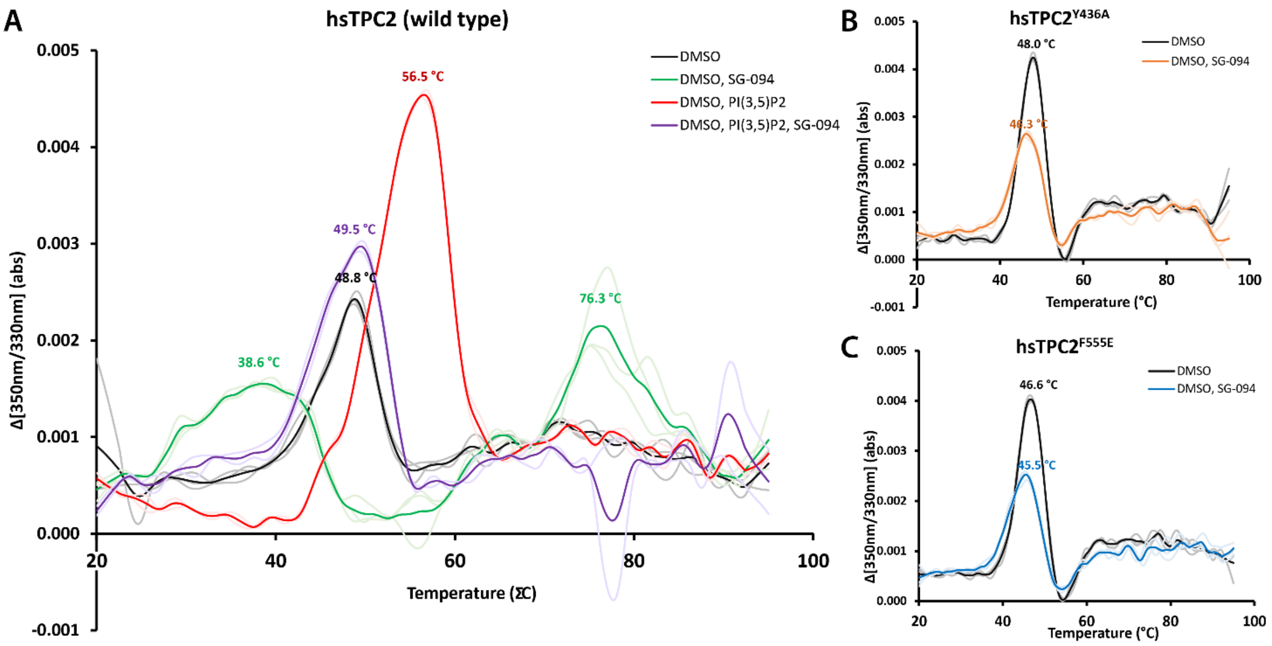

Supplementary Figure S2. Loss of *Hs*TPC2 stabilization by SG-094 for binding site mutants Y436A and F555E characterized in thermal shift experiments, related to Figures 4 and 5.

A– C) Thermal shift assay of *Hs*TPC2^{WT} (**A**) shows significant differences in melting profiles between apo (black trace) and SG-094-bound (green trace), suggesting that the compound binding alters the melting behavior of purified *Hs*TPC2. It also shows that the PI(3,5)P_2-mediated increase in thermostability (red trace) reversed to apo-like level (purple). In contrast, *Hs*TPC2^{Y436A} (**B**) and *Hs*TPC2^{F555E} (**C**) show little difference between apo state samples and SG-094-bound samples, indicating significantly reduced binding of the antagonist to *Hs*TPC2 with these mutations.

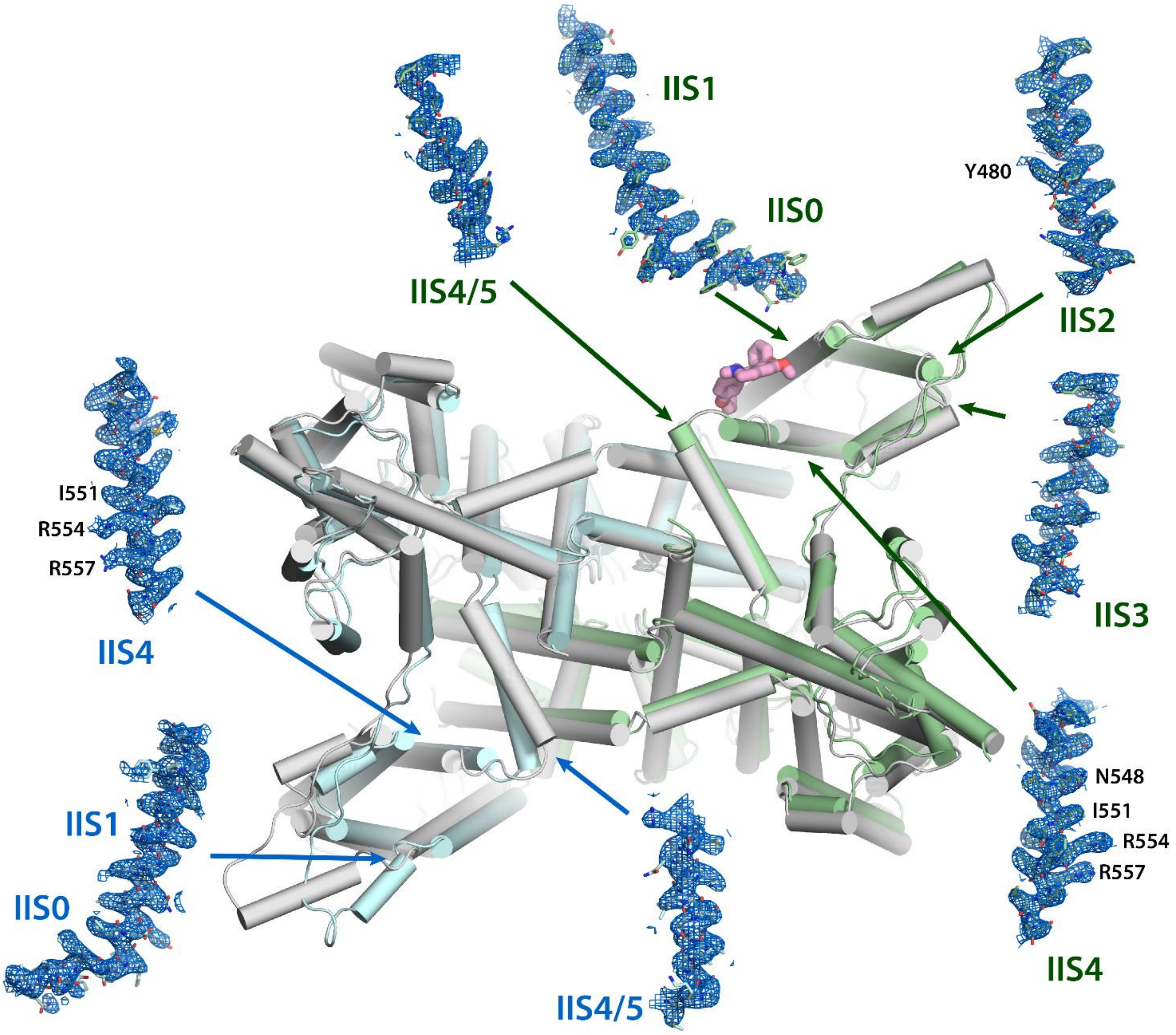

Supplementary Figure S3. Electron map features for key sites for the structure of HsTPC2 in complex with *(S)*-SG-094, related to Figures 1, 2, 6 and 7.

All electrostatic potential (ESP) map features have been contoured at 6.0 σ for consistency.

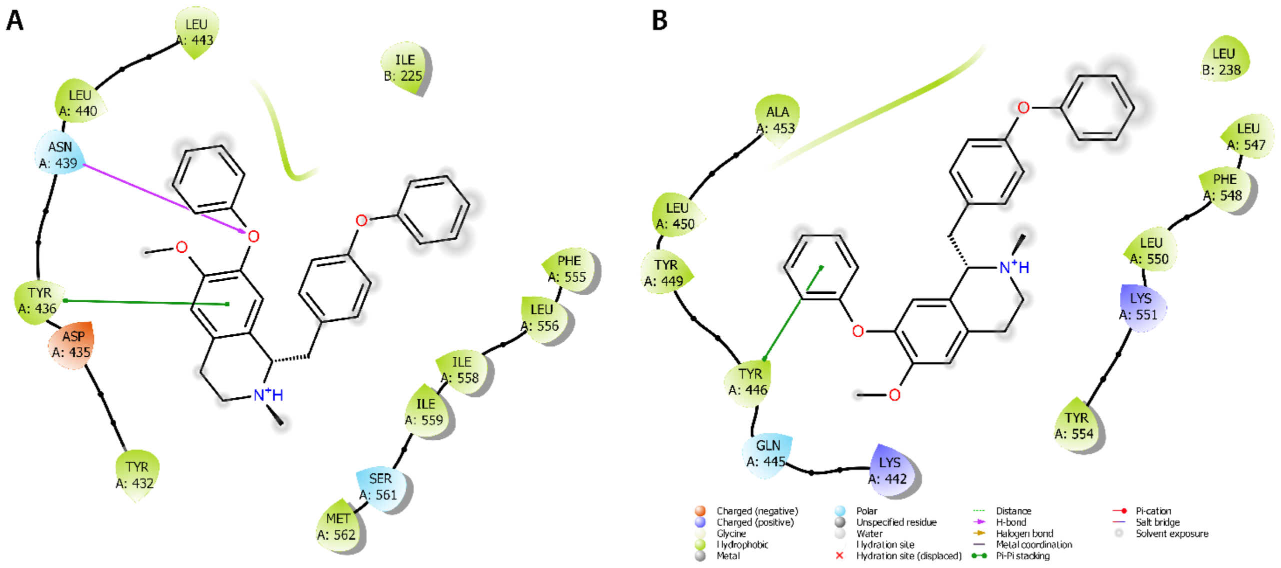

Supplementary Figure S4. Comparison of SG-094 interactions with *Hs*TPC2 to putative interactions of the antagonist bound to *Mm*TPC1, related to Figure 1.

A) Schematic of molecular interactions between SG-094 antagonist and residues in *Hs*TPC2 as inferred from the cryo-EM structure. **B)** Schematic of putative molecular interactions between SG-094 antagonist and the corresponding residues in *Hs*TPC1 as inferred from docking into the analogous binding site of *Mm*TPC1 (PDB ID: 6C96).

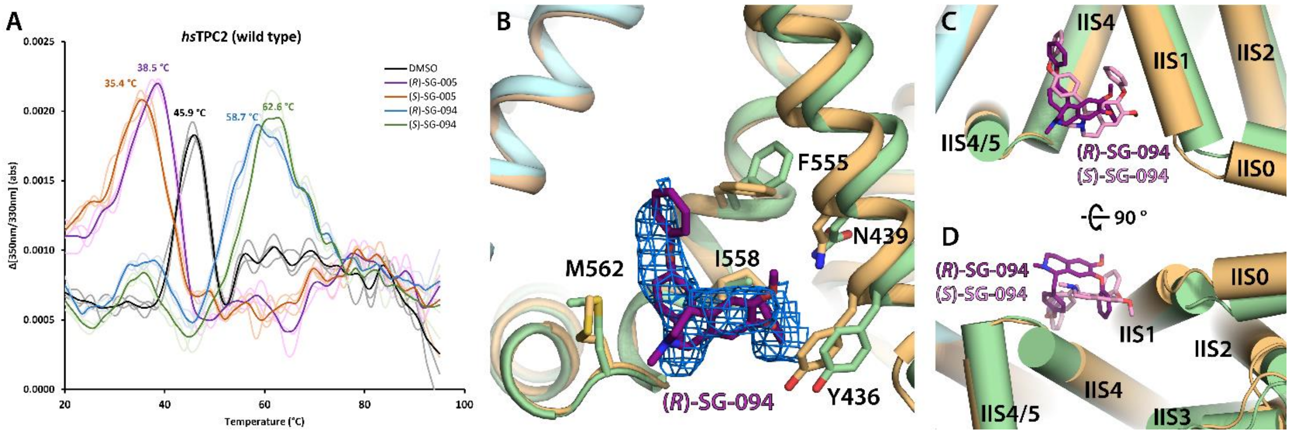

Supplementary Figure S5. Comparison of *(S)*-SG-094 and *(R)*-SG-094 interactions with *Hs*TPC2 showing similar biophysical properties and binding location, related to Figure 1.

A) Thermal shift experiments of SG-094 and SG-005 enantiomers show similar levels of stabilisation between the enantiomers of each compound. **B)** Cryo-EM electrostatic potential map of *Hs*TPC2 with *(R)*-SG-094 (σ=4.5) shows small compound feature at the same binding site as *(S)*-SG-094, however the level of fit is not good enough for modelling with confidence. Key residues are positioned similarly between *(R)*-SG-094 (light brown) and *(S)*-SG-094 (light green) bound *Hs*TPC2, suggesting similar effects of the compounds on the protein conformation. **C)** Comparison of *(S)*-SG-094 and *(R)*-SG-094 models shows near-identical binding site but with differences in binding mode.

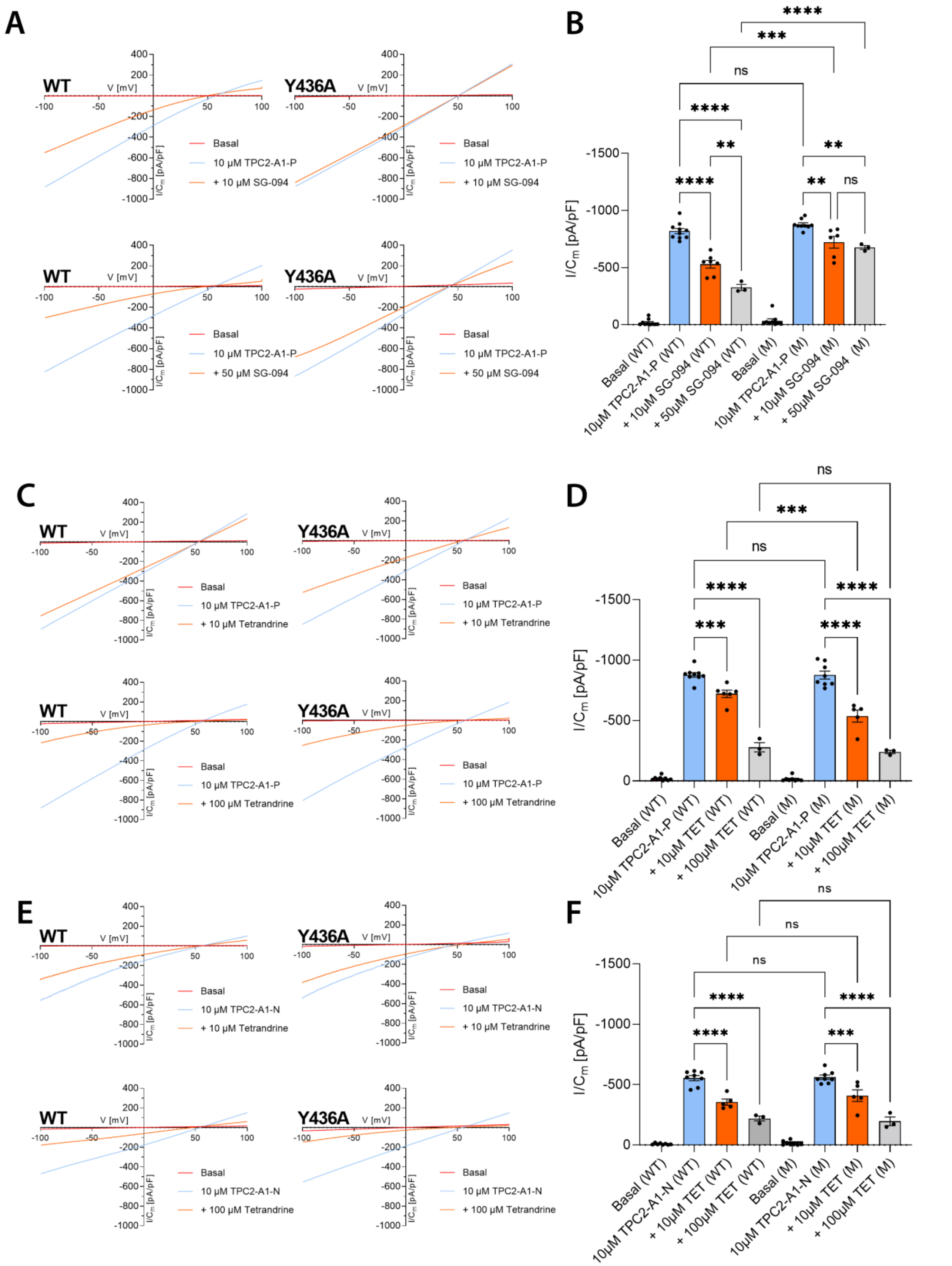

Supplementary Figure S6. TPC2-A1-P or TPC2-A1-N evoked hTPC2 current inhibited by SG-094 or tetrandrine for binding site mutant Y436A in whole cell patch-clamp recordings, related to Figures 4 and 5.

Representative current density-voltage (I/Cm-V) relation of transiently expressed, plasma membrane-targeted *Hs*TPC2$^{L11A/L12A}$-eYFP variants, WT (A, C and E – left) and Y436A mutant (A,

C and E – right). Channels were activated by application of TPC2-A1-P or TPC2-A1-N (10 µM, blue traces), followed by application of the TPC antagonists SG-094 (A; 10 µM and 50 µM, orange traces) tetrandrine (C, E; 10µM and 100 µM, orange traces). Basal current density was depicted with red color. Statistical analysis of experiments is shown in B, D, and F, with each dot representing mean of 5 – 10 technical replicate measurements (mean ± SEM; n = number of dots on the graph; one-way ANOVA, Tukey's post hoc test using GraphPad Prism 9.0.2, **** $p<0.0001$, *** $p<0.001$, ** $p<0.01$, n.s. - not significant).

Supplementary Table S1, related to STAR Methods (Cryo-EM Sample Preparation, Data Collection and Processing; Model Building and Refinement; Quantification and Statistical Analysis). Data table for cryo-EM data collection, refinement and validation statistics of *(S)*-SG-094-bound *Hs*TPC2 and *(R)*-SG-094-bound *Hs*TPC2

	***Hs*TPC2 with *(S)*-SG-094**	***Hs*TPC2 with *(R)*-SG-094**
**Microscope**	Titan Krios	Titan Krios
**Detector**	K2	K2
**Voltage (kV)**	300	300
**Magnification**	105,000	105,000
**Collection mode**	Counting, superresolution	Counting, superresolution
**Electron exposure (e/Å ^{2})**	38	42
**Number of frames**	31	36
**Pixel size (Å)**	0.4145	0.4145
**Defocus range (µm; steps)**	-0.8 to -2.4 (0.2)	-0.8 to -2.4 (0.2)
**Number of movies**	9,924	5,848
**Initial Number of particles**	2,508,787	2,290,202
**Number of particles after 2D classification**	751,050	198,508
**Symmetry**	C1	C1
**Number of particles used for 3D refinement**	109,417	99,232
**Map resolution (Å ; FSC threshold = 0.143)**	3.0	3.6
**Resolution range (Å)**	2.6 – 43.8	3.1 – 45.2
**Map sharpening B-factor (Å ^{2})**	80.9	94.9
**Model resolution (Å ; FSC threshold = 0.5)**	3.2	
**Non-hydrogen atoms**	10,749	
**Protein residues**	1,273	
**Ligands**	14	
**R.M.S.D**		
Bond lengths (Å)	0.002	
Bond angles (°)	0.468	
**Validation**		
Molprobity score	1.69	
Clash score	5.76	
Rotamer outliers (%)	1.53	
**Ramachandran plot**		
Favoured (%)	96.42	
Allowed (%)	3.58	
Disallowed (%)	0	
**EMDB Code**	EMD-17197	EMD-19108
**PDB Code**	8OUO	

Supplementary Table S2, related to STAR Methods (Key Resources Table). DNA sequences for synthetic codon-optimised genes

Gene	Sequence
*Hs*TPC2	ATGGCAGAACCTCAAGCAGAAAGCGAACCAGCAGCAGGTGGAGCAAGGGGTGGCGGCGGTGA<br>TTGGCCTGCTGGTTTGACCACTTACCGCAGCATCCAAGTCGGCCCTGGTGCCGCGGCCAGGTGG<br>GACCTCTGCATTGATCAGGCTGTGGTCTTCATCGAAGATGCTATTCAGTACCGCTCCATCAACC<br>ACCGGGTGGATGCCAGCTCGATGTGGCTTTACCGACGGTATTACTCGAACGTATGCCAACGGA<br>CTTTGAGCTTCACCATCTTCTTGATCCTGTTTTTGGCTTTTATCGAGACCCCATCCTCACTCACC<br>AGCACGGCGGACGTGCGCTACCGCGCTGCCCCCTGGGAGCCGCCCTGCGGCCTGACCGAGAGT<br>GTCGAGGTGCTCTGCCTGCTGGTCTTTGCGGCCGACCTCTCTGTGAAGGGTTACCTGTTCGGGT<br>GGGCCCATTTCCAGAAAAACCTTTGGCTGCTGGGCTACCTCGTGGTGCTGGTGGTGTCTCTGGT<br>GGACTGGACCGTGTCCCTGAGTCTCGTGTGTCATGAGCCCCTGCGGATCCGCCGGCTTCTCCGT<br>CCCTTCTTCCTGCTGCAGAACTCCTCTATGATGAAGAAGACCTTGAAATGCATCCGCTGGTCGC<br>TGCCGGAAATGGCCAGCGTCGGGCTGCTGCTGGCCATCCACCTGTGCCTCTTCACCATGTTCGG<br>AATGCTGCTGTTCGCTGGTGGGAAGCAGGATGATGGGCAGGACAGGGAGAGGCTGACCTACTT<br>CCAGAACCTGCCTGAGTCTCTGACTTCCCTCCTGGTGCTGCTGACCACGGCCAACAACCCCGAT<br>GTGATGATTCCTGCGTATTCCAAGAACCGGGCCTATGCCATCTTCTTCATAGTCTTCACTGTGAT<br>AGGAAGCCTGTTTCTGATGAACCTGCTGACAGCCATCATCTACAGTCAGTTCCGGGGCTACCTG<br>ATGAAATCTCTCCAGACCTCGCTGTTTCGGAGGCGGCTGGGAACCCGGGCTGCCTTTGAAGTCC<br>TATCCTCCATGGTGGGGGAGGGAGGAGCCTTCCCTCAGGCAGTTGGGGTGAAGCCCCAGAACT<br>TGCTGCAGGTGCTTCAGAAGGTCCAGCTGGACAGCTCCCACAAACAGGCCATGATGGAGAAGG<br>TGCGTTCCTACGGCAGTGTTCTGCTGTCAGCTGAGGAGTTTCAGAAGCTCTTCAACGAGCTTGA<br>CAGAAGTGTGGTTAAAGAGCACCCGCCGAGGCCCGAGTACCAGTCTCCGTTTCTGCAGAGCGC<br>CCAGTTCCTCTTCGGCCACTACTACTTTGACTACCTGGGGAACCTCATCGCCCTGGCAAACCTG<br>GTGTCCATTTGCGTGTTCCTGGTGCTGGATGCAGATGTGCTGCCTGCTGAGCGTGATGACTTCA<br>TCCTGGGGATTCTCAACTGCGTCTTCATTGTGTACTACCTGTTGGAGATGCTGCTCAAGGTCTTT<br>GCCCTGGGCCTGCGAGGGTACCTGTCCTACCCCAGCAACGTGTTTGACGGGCTCCTCACCGTTG<br>TCCTGCTGGTTTTGGAGATCTCAACTCTGGCTGTGTACCGATTGCCACACCCAGGCTGGAGGCC<br>GGAGATGGTGGGCCTGCTGTCGCTGTGGGACATGACCCGCATGCTGAACATGCTCATCGTGTTC<br>CGCTTCCTGCGTATCATCCCCAGCATGAAGCCGATGGCCGTGGTGGCCAGTACCGTCCTGGGCC<br>TGGTGCAGAACATGCGTGCTTTTGGCGGGATCCTGGTGGTGGTCTACTACGTATTTGCCATCAT<br>TGGGATCAACTTGTTTAGAGGCGTCATTGTGGCTCTTCCTGGAAACAGCAGCCTGGCCCCTGCC<br>AATGGCTCGGCGCCCTGTGGGAGCTTCGAGCAGCTGGAGTACTGGGCCAACAACTTCGATGAC<br>TTTGCGGCTGCCCTGGTCACTCTGTGGAACTTGATGGTGGTGAACAACTGGCAGGTGTTTCTGG<br>ATGCATATCGGCGCTACTCAGGCCCGTGGTCCAAGATCTATTTTGTATTGTGGTGGCTGGTGTC<br>GTCTGTCATCTGGGTCAACCTGTTTCTGGCCCTGATTCTGGAGAACTTCCTTCACAAGTGGGAC<br>CCCCGCAGCCACCTGCAGCCCCTTGCTGGGACCCCAGAGGCCACCTACCAGATGACTGTGGAG<br>CTCCTGTTCAGGGATATTCTGGAGGAGCCCGAGGAGGATGAGCTCACAGAGAGGCTGAGCCAG<br>CACCCGCACCTGTGGCTGTGCAGGtga
*Hs*TPC2^{Y436A}	ATGGCAGAACCTCAAGCAGAAAGCGAACCAGCAGCAGGTGGAGCAAGGGGTGGCGGCGGTGA<br>TTGGCCTGCTGGTTTGACCACTTACCGCAGCATCCAAGTCGGCCCTGGTGCCGCGGCCAGGTGG<br>GACCTCTGCATTGATCAGGCTGTGGTCTTCATCGAAGATGCTATTCAGTACCGCTCCATCAACC<br>ACCGGGTGGATGCCAGCTCGATGTGGCTTTACCGACGGTATTACTCGAACGTATGCCAACGGA<br>CTTTGAGCTTCACCATCTTCTTGATCCTGTTTTTGGCTTTTATCGAGACCCCATCCTCACTCACC<br>AGCACGGCGGACGTGCGCTACCGCGCTGCCCCCTGGGAGCCGCCCTGCGGCCTGACCGAGAGT

	GTCGAGGTGCTCTGCCTGCTGGTCTTTGCGGCCGACCTCTCTGTGAAGGGTTACCTGTTCGGGT<br>GGGCCCATTTCCAGAAAAACCTTTGGCTGCTGGGCTACCTCGTGGTGCTGGTGGTGTCTCTGGT<br>GGACTGGACCGTGTCCCTGAGTCTCGTGTGTCATGAGCCCCTGCGGATCCGCCGGCTTCTCCGT<br>CCCTTCTTCCTGCTGCAGAACTCCTCTATGATGAAGAAGACCTTGAAATGCATCCGCTGGTCGC<br>TGCCGGAAATGGCCAGCGTCGGGCTGCTGCTGGCCATCCACCTGTGCCTCTTCACCATGTTCGG<br>AATGCTGCTGTTCGCTGGTGGGAAGCAGGATGATGGGCAGGACAGGGAGAGGCTGACCTACTT<br>CCAGAACCTGCCTGAGTCTCTGACTTCCCTCCTGGTGCTGCTGACCACGGCCAACAACCCCGAT<br>GTGATGATTCCTGCGTATTCCAAGAACCGGGCCTATGCCATCTTCTTCATAGTCTTCACTGTGAT<br>AGGAAGCCTGTTTCTGATGAACCTGCTGACAGCCATCATCTACAGTCAGTTCCGGGGCTACCTG<br>ATGAAATCTCTCCAGACCTCGCTGTTTCGGAGGCGGCTGGGAACCCGGGCTGCCTTTGAAGTCC<br>TATCCTCCATGGTGGGGGAGGGAGGAGCCTTCCCTCAGGCAGTTGGGGTGAAGCCCCAGAACT<br>TGCTGCAGGTGCTTCAGAAGGTCCAGCTGGACAGCTCCCACAAACAGGCCATGATGGAGAAGG<br>TGCGTTCCTACGGCAGTGTTCTGCTGTCAGCTGAGGAGTTTCAGAAGCTCTTCAACGAGCTTGA<br>CAGAAGTGTGGTTAAAGAGCACCCGCCGAGGCCCGAGTACCAGTCTCCGTTTCTGCAGAGCGC<br>CCAGTTCCTCTTCGGCCACTACTACTTTGACGCCCTGGGGAACCTCATCGCCCTGGCAAACCTG<br>GTGTCCATTTGCGTGTTCCTGGTGCTGGATGCAGATGTGCTGCCTGCTGAGCGTGATGACTTCA<br>TCCTGGGGATTCTCAACTGCGTCTTCATTGTGTACTACCTGTTGGAGATGCTGCTCAAGGTCTTT<br>GCCCTGGGCCTGCGAGGGTACCTGTCCTACCCCAGCAACGTGTTTGACGGGCTCCTCACCGTTG<br>TCCTGCTGGTTTTGGAGATCTCAACTCTGGCTGTGTACCGATTGCCACACCCAGGCTGGAGGCC<br>GGAGATGGTGGGCCTGCTGTCGCTGTGGGACATGACCCGCATGCTGAACATGCTCATCGTGTTC<br>CGCTTCCTGCGTATCATCCCCAGCATGAAGCCGATGGCCGTGGTGGCCAGTACCGTCCTGGGCC<br>TGGTGCAGAACATGCGTGCTTTTGGCGGGATCCTGGTGGTGGTCTACTACGTATTTGCCATCAT<br>TGGGATCAACTTGTTTAGAGGCGTCATTGTGGCTCTTCCTGGAAACAGCAGCCTGGCCCCTGCC<br>AATGGCTCGGCGCCCTGTGGGAGCTTCGAGCAGCTGGAGTACTGGGCCAACAACTTCGATGAC<br>TTTGCGGCTGCCCTGGTCACTCTGTGGAACTTGATGGTGGTGAACAACTGGCAGGTGTTTCTGG<br>ATGCATATCGGCGCTACTCAGGCCCGTGGTCCAAGATCTATTTTGTATTGTGGTGGCTGGTGTC<br>GTCTGTCATCTGGGTCAACCTGTTTCTGGCCCTGATTCTGGAGAACTTCCTTCACAAGTGGGAC<br>CCCCGCAGCCACCTGCAGCCCCTTGCTGGGACCCCAGAGGCCACCTACCAGATGACTGTGGAG<br>CTCCTGTTCAGGGATATTCTGGAGGAGCCCGAGGAGGATGAGCTCACAGAGAGGCTGAGCCAG<br>CACCCGCACCTGTGGCTGTGCAGGtga
*Hs*TPC2^{F555E}	ATGGCAGAACCTCAAGCAGAAAGCGAACCAGCAGCAGGTGGAGCAAGGGGTGGCGGCGGTGA<br>TTGGCCTGCTGGTTTGACCACTTACCGCAGCATCCAAGTCGGCCCTGGTGCCGCGGCCAGGTGG<br>GACCTCTGCATTGATCAGGCTGTGGTCTTCATCGAAGATGCTATTCAGTACCGCTCCATCAACC<br>ACCGGGTGGATGCCAGCTCGATGTGGCTTTACCGACGGTATTACTCGAACGTATGCCAACGGA<br>CTTTGAGCTTCACCATCTTCTTGATCCTGTTTTTGGCTTTTATCGAGACCCCATCCTCACTCACC<br>AGCACGGCGGACGTGCGCTACCGCGCTGCCCCCTGGGAGCCGCCCTGCGGCCTGACCGAGAGT<br>GTCGAGGTGCTCTGCCTGCTGGTCTTTGCGGCCGACCTCTCTGTGAAGGGTTACCTGTTCGGGT<br>GGGCCCATTTCCAGAAAAACCTTTGGCTGCTGGGCTACCTCGTGGTGCTGGTGGTGTCTCTGGT<br>GGACTGGACCGTGTCCCTGAGTCTCGTGTGTCATGAGCCCCTGCGGATCCGCCGGCTTCTCCGT<br>CCCTTCTTCCTGCTGCAGAACTCCTCTATGATGAAGAAGACCTTGAAATGCATCCGCTGGTCGC<br>TGCCGGAAATGGCCAGCGTCGGGCTGCTGCTGGCCATCCACCTGTGCCTCTTCACCATGTTCGG<br>AATGCTGCTGTTCGCTGGTGGGAAGCAGGATGATGGGCAGGACAGGGAGAGGCTGACCTACTT<br>CCAGAACCTGCCTGAGTCTCTGACTTCCCTCCTGGTGCTGCTGACCACGGCCAACAACCCCGAT<br>GTGATGATTCCTGCGTATTCCAAGAACCGGGCCTATGCCATCTTCTTCATAGTCTTCACTGTGAT<br>AGGAAGCCTGTTTCTGATGAACCTGCTGACAGCCATCATCTACAGTCAGTTCCGGGGCTACCTG<br>ATGAAATCTCTCCAGACCTCGCTGTTTCGGAGGCGGCTGGGAACCCGGGCTGCCTTTGAAGTCC

	TATCCTCCATGGTGGGGGAGGGAGGAGCCTTCCCTCAGGCAGTTGGGGTGAAGCCCCAGAACT<br>TGCTGCAGGTGCTTCAGAAGGTCCAGCTGGACAGCTCCCACAAACAGGCCATGATGGAGAAGG<br>TGCGTTCCTACGGCAGTGTTCTGCTGTCAGCTGAGGAGTTTCAGAAGCTCTTCAACGAGCTTGA<br>CAGAAGTGTGGTTAAAGAGCACCCGCCGAGGCCCGAGTACCAGTCTCCGTTTCTGCAGAGCGC<br>CCAGTTCCTCTTCGGCCACTACTACTTTGACTACCTGGGGAACCTCATCGCCCTGGCAAACCTG<br>GTGTCCATTTGCGTGTTCCTGGTGCTGGATGCAGATGTGCTGCCTGCTGAGCGTGATGACTTCA<br>TCCTGGGGATTCTCAACTGCGTCTTCATTGTGTACTACCTGTTGGAGATGCTGCTCAAGGTCTTT<br>GCCCTGGGCCTGCGAGGGTACCTGTCCTACCCCAGCAACGTGTTTGACGGGCTCCTCACCGTTG<br>TCCTGCTGGTTTTGGAGATCTCAACTCTGGCTGTGTACCGATTGCCACACCCAGGCTGGAGGCC<br>GGAGATGGTGGGCCTGCTGTCGCTGTGGGACATGACCCGCATGCTGAACATGCTCATCGTGTTC<br>CGCGCCCTGCGTATCATCCCCAGCATGAAGCCGATGGCCGTGGTGGCCAGTACCGTCCTGGGC<br>CTGGTGCAGAACATGCGTGCTTTTGGCGGGATCCTGGTGGTGGTCTACTACGTATTTGCCATCA<br>TTGGGATCAACTTGTTTAGAGGCGTCATTGTGGCTCTTCCTGGAAACAGCAGCCTGGCCCCTGC<br>CAATGGCTCGGCGCCCTGTGGGAGCTTCGAGCAGCTGGAGTACTGGGCCAACAACTTCGATGA<br>CTTTGCGGCTGCCCTGGTCACTCTGTGGAACTTGATGGTGGTGAACAACTGGCAGGTGTTTCTG<br>GATGCATATCGGCGCTACTCAGGCCCGTGGTCCAAGATCTATTTTGTATTGTGGTGGCTGGTGT<br>CGTCTGTCATCTGGGTCAACCTGTTTCTGGCCCTGATTCTGGAGAACTTCCTTCACAAGTGGGA<br>CCCCCGCAGCCACCTGCAGCCCCTTGCTGGGACCCCAGAGGCCACCTACCAGATGACTGTGGA<br>GCTCCTGTTCAGGGATATTCTGGAGGAGCCCGAGGAGGATGAGCTCACAGAGAGGCTGAGCCA<br>GCACCCGCACCTGTGGCTGTGCAGGtga
